# Applications of Fourier Transform-Infrared spectroscopy in microbial cell biology and environmental microbiology: advances, challenges, and future perspectives

**DOI:** 10.3389/fmicb.2023.1304081

**Published:** 2023-11-21

**Authors:** Amin Kassem, Lana Abbas, Oliver Coutinho, Somie Opara, Hawraa Najaf, Diana Kasperek, Keshav Pokhrel, Xiaohua Li, Sonia Tiquia-Arashiro

**Affiliations:** ^1^Department of Natural Sciences, University of Michigan-Dearborn, Dearborn, MI, United States; ^2^Department of Mathematics and Statistics, University of Michigan-Dearborn, Dearborn, MI, United States

**Keywords:** Fourier transform infrared spectroscopy, cellular structures, metabolic activities, bacteria, microbial stress, cell membrane, population dynamics

## Abstract

Microorganisms play pivotal roles in shaping ecosystems and biogeochemical cycles. Their intricate interactions involve complex biochemical processes. Fourier Transform-Infrared (FT-IR) spectroscopy is a powerful tool for monitoring these interactions, revealing microorganism composition and responses to the environment. This review explores the diversity of applications of FT-IR spectroscopy within the field of microbiology, highlighting its specific utility in microbial cell biology and environmental microbiology. It emphasizes key applications such as microbial identification, process monitoring, cell wall analysis, biofilm examination, stress response assessment, and environmental interaction investigation, showcasing the crucial role of FT-IR in advancing our understanding of microbial systems. Furthermore, we address challenges including sample complexity, data interpretation nuances, and the need for integration with complementary techniques. Future prospects for FT-IR in environmental microbiology include a wide range of transformative applications and advancements. These include the development of comprehensive and standardized FT-IR libraries for precise microbial identification, the integration of advanced analytical techniques, the adoption of high-throughput and single-cell analysis, real-time environmental monitoring using portable FT-IR systems and the incorporation of FT-IR data into ecological modeling for predictive insights into microbial responses to environmental changes. These innovative avenues promise to significantly advance our understanding of microorganisms and their complex interactions within various ecosystems.

## 1. Introduction

Fourier Transform-Infrared (FTIR) spectroscopy has emerged as an exceptionally versatile and indispensable tool, revolutionizing molecular analysis across a spectrum of scientific domains, including microbiology (Beekes et al., 2007; Baker et al., 2014; Zarnowiec et al., 2015; Nandiyanto et al., 2019; He et al., 2022; Koczoń et al., 2023). This analytical technique operates on the principle that molecules absorb specific frequencies of infrared light, which correspond to the vibrational frequencies of their chemical bonds (Griffiths, 1983; Nandiyanto et al., 2019; He et al., 2022; Lilo et al., 2022; Weber et al., 2023). The absorption produces a characteristic spectrum that can be used to identify the functional groups and molecular structures present in a sample.

FTIR spectroscopy has been a valuable tool in various biological studies across different scientific areas. In the field of microbiology, it has significantly aided in the rapid and accurate identification of microorganisms (Helm et al., 1991; Wenning and Scherer, 2013; Corte et al., 2014; Câmara et al., 2020; Feng et al., 2020; Brito and Lourenço, 2021; Smirnova et al., 2022; Kamnev et al., 2023; Tata et al., 2023; Tessaro et al., 2023), contributing to timely infection diagnosis and the implementation of appropriate treatment methods. This technique has also allowed for in-depth analyses of microbial structures, metabolic activities, and responses to environmental changes, leading to a better understanding of microbial physiology and behavior (Corte et al., 2014; Câmara et al., 2020; Smirnova et al., 2022; Tessaro et al., 2023). Moreover, FTIR spectroscopy has played a crucial role in monitoring and evaluating the dynamics of microbial communities in different environments (Kamnev et al., 2023; Marques et al., 2023). This has contributed to the management of water quality, assessment of ecosystem health, and the detection of microbial pollution. In the context of metal-pollutant bioremediation, FTIR has enabled the comprehensive analysis of how microorganisms interact with metal contaminants (Pagnucco et al., 2023). This has helped in understanding the mechanisms involved in metal sequestration, transformation, and detoxification. Furthermore, in the domain of organic pollutant bioremediation, FTIR has served as a valuable tool in investigating the interactions between microorganisms and organic contaminants. It has provided insights into the biochemical transformations and degradation pathways involved in the bioremediation process (Aziz et al., 2020).

This review explores the diverse applications of FT-IR spectroscopy in microbiology, with a particular emphasis on its use in microbial cell biology and environmental microbiology. While previous reviews on FT-IR have provided valuable insights into the general principles and applications of the technology, our study seeks to differentiate itself by focusing on its applications in microbial cell biology and environmental microbiology. Additionally, this review addresses the limitations of FT-IR in these disciplines and the challenges that researchers encounter when practically employing FT-IR technology. The review also outlines potential avenues for future research and development, laying the groundwork for further advancements in the field of microbiology and FT-IR technology.

## 2. Basic principles of FTIR

FT-IR spectroscopy is based on the principle that molecules absorb specific frequencies of infrared light, corresponding to the vibrational frequencies of their chemical bonds. When a sample is exposed to infrared light, the molecules within the sample absorb this light at characteristic frequencies, causing the bonds within the molecules to vibrate. Different types of bonds, such as C-H, O-H, and N-H bonds, have distinct vibrational frequencies, leading to unique patterns in the absorption of infrared light. By measuring the intensity of the absorbed light at various wavelengths, an FT-IR spectrometer produces a characteristic spectrum for the sample. This spectrum can be used to identify the functional groups and molecular structures present in the sample, essentially providing a “fingerprint” that can be used for qualitative and quantitative analysis. FT-IR spectroscopy can be used to study a diverse array of specimens, including solids, liquids, and gases, and it has applications across various scientific domains, including chemistry, physics, materials science, and biology (Griffiths, 1983; Koczoń et al., 2023).

**Figure 1:**
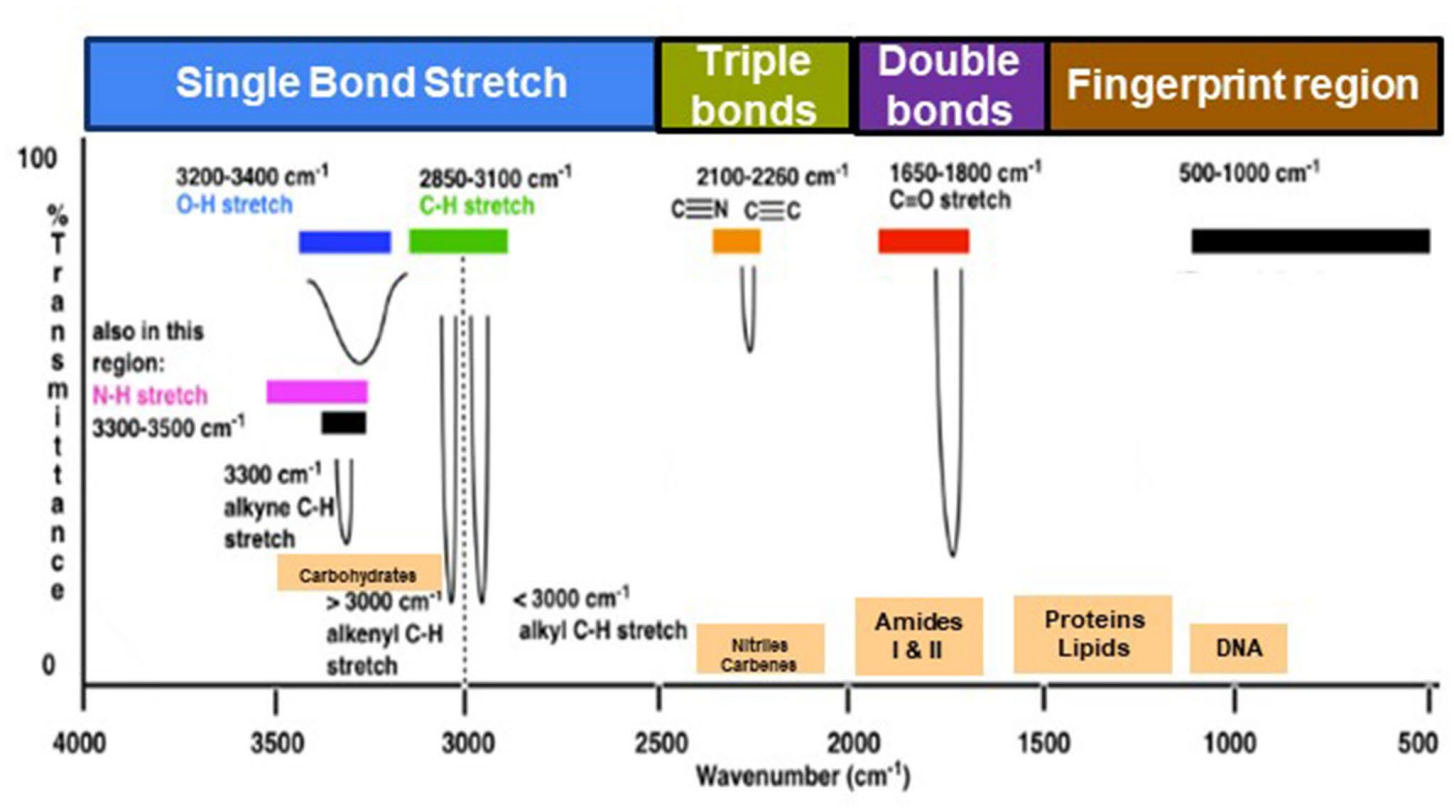
Typical infrared values for various types of bonds. The region 500–1,500 cm^−1^, which is in the mid-IR region, is called the fingerprint region and provides molecular fingerprints unique to specific compounds. Reproduced from Master Organic Chemistry (https://www.masterorganicchemistry.com/2016/11/23/quick_analysis_of_ir_spectra/) (accessed on 28 September 2023), with kind permission from James Ashenhurst.

The range of IR radiation encompasses electromagnetic radiation with frequencies between 14,300 and 20 cm^−1^, with the most significant vibrational frequencies for most molecules falling within the mid IR spectrum, ranging between 4,000 and 400 cm^−1^ (Griffiths, 1983). Within this specific range, there are four different regions: (1) the single bond region (2,500–4,000 cm^−1^), (2) the triple bond region (2,000–2,500 cm^−1^), (3) the double bond region (1,500–2,000 cm^−1^), and (4) the fingerprint region (600–1,500 cm^−1^) (Figure 1). Table 1 shows some common peaks observed in FT-IR spectra, along with their corresponding functional groups and reference wavenumbers. Far-and near-IR ranges are less frequently employed, primarily because these regions register overtone (secondary) vibrations and combination vibrations, making them analytically challenging to study and interpret (Nandiyanto et al., 2019; Pirutin et al., 2023).

**Table 1:** Possible assignment of some bands frequently found in microbial IR spectra (peak frequencies have been obtained from the second derivative spectra).

Assignment	Frequency (cm^−1^)
C-H stretching peaks	
Alkane C-H stretching	2,850–2,960
Alkene C-H stretching	3,010–3,100
Alkyne C-H stretching	3,300
O-H and N-H stretching peaks	
Alcohols and phenols O-H stretching	3,200–3,600
Carboxylic acids O-H stretching	2,500–3,300
Amines N-H stretching	3,300–3,500
C=O stretching peaks	
Ketones and aldehydes C=O stretching	1,680–1,750
Carboxylic acids C=O stretching	1,700–1,750
Amides C=O stretching	1,630–1,690
C-N stretching peaks	
Aromatic C-N stretching	1,300–1,350
Aliphatic C-N stretching	1,000–1,300
C=C stretching peaks	
Alkene C=C stretching	1,620–1,680
Aromatic C=C stretching	1,450–1,600
N-H bending peaks	
Amines N-H bending	1,560–1,640
Amides N-H bending	1,550–1,670
C-H bending peaks	
Alkanes C-H bending	1,370–1,470
Alkenes C-H bending	960–1,200
O-H bending peaks	
Alcohols and phenols O-H bending	1,350–1,450
Fingerprint region peaks	700–900

Adapted from Smith (1998) and Silverstein et al. (2014).

Within the mid IR spectrum, researchers commonly use five distinct spectral windows (Figure 1). The first window, ranging from 3,000 to 2,800 cm^−1^, is primarily influenced by specific functional groups like membrane fatty acids and certain amino acid side-chain vibrations, dominated by C-H stretching vibrations of CH₃ and CH₂ functional groups. The second window, between 1,800 and 1,500 cm^−1^, is affected by amide I and amide II groups in proteins and peptides, showcasing intense peaks providing comprehensive insights into protein structure, along with vibrations of ester functional groups in lipids and nucleic acid absorptions. The third window, spanning 1,500 to 1,200 cm^−1^, represents a mixed region influenced by proteins, fatty acids, and compounds with phosphate groups, affected by CH₂ and CH₃ bending modes. The fourth window, from 1,200 to 900 cm^−1^, is characterized by symmetric stretching vibrations of PO₂^−^ groups in nucleic acids, along with vibrations related to carbohydrates, polysaccharides, and nucleic acids. Lastly, the fifth window, between 900 and 700 cm^−1^, known as the true fingerprint region, demonstrates subtle spectral patterns arising from vibrations of aromatic rings in specific amino acids and nucleotides.

The spectrum’s peaks serve as identifiers for functional groups in both organic and inorganic compounds, utilizing their characteristic absorption bands within the infrared region of the electromagnetic spectrum (Griffiths, 1983; El-Azazy et al., 2021). These functional groups, linked to specific vibrational modes resulting from atomic movements within the groups (Griffiths, 1983; Smith, 1998; El-Azazy et al., 2021), encompass stretching, bending, and combination bands. Expressing the frequencies of these vibrations in wavenumbers (cm^−1^) enables the identification of particular functional groups. For instance, the C=O stretching vibration of a carbonyl group, typically found in ketones and aldehydes, appears around 1,700–1,750 cm^−1^ (Table 1). Meanwhile, the O-H stretching vibration of alcohols typically appears around 3,200–3,600 cm^−1^ (Table 1). By comparing the absorption bands’ positions and intensities in the sample’s spectrum to known reference spectra, analysts can determine the presence of specific functional groups in the sample (Silverstein et al., 2014). Notably, complex and distinctive patterns within the ~1,500–400 cm^−1^ region are highly specific to the compound’s molecular structure. However, peak positions may vary due to factors such as the molecular environment, sample preparation, and instrument settings, underscoring the importance of consulting reliable databases and literature for accurate peak assignments (Griffiths, 1983; Silverstein et al., 2014; He et al., 2022).

## 3. Types of FT-IR used in microbiology

In microbiology, various forms of Fourier Transform Infrared (FT-IR) spectroscopy serve different research purposes. Transmission FT-IR involves transmitting infrared light through a sample to measure the transmitted light and absorption spectrum, proving valuable for analyzing solid samples like microbial cell walls or biofilm components. Attenuated Total Reflection (ATR) FT-IR is used for liquid and solid samples unsuitable for transmission FT-IR, enabling analysis without extensive sample preparation by measuring the sample’s infrared spectrum in contact with an ATR crystal. Micro-FTIR Spectroscopy allows microscopic analysis of small sample areas, providing high spatial resolution and detailed information about the molecular composition of specific microorganisms or microbial components within a sample. Diffuse Reflectance Fourier Transform Infrared (DRIFT) spectroscopy is ideal for analyzing powdered or granulated samples, including microbial samples, without demanding extensive sample preparation.

**Figure 2:**
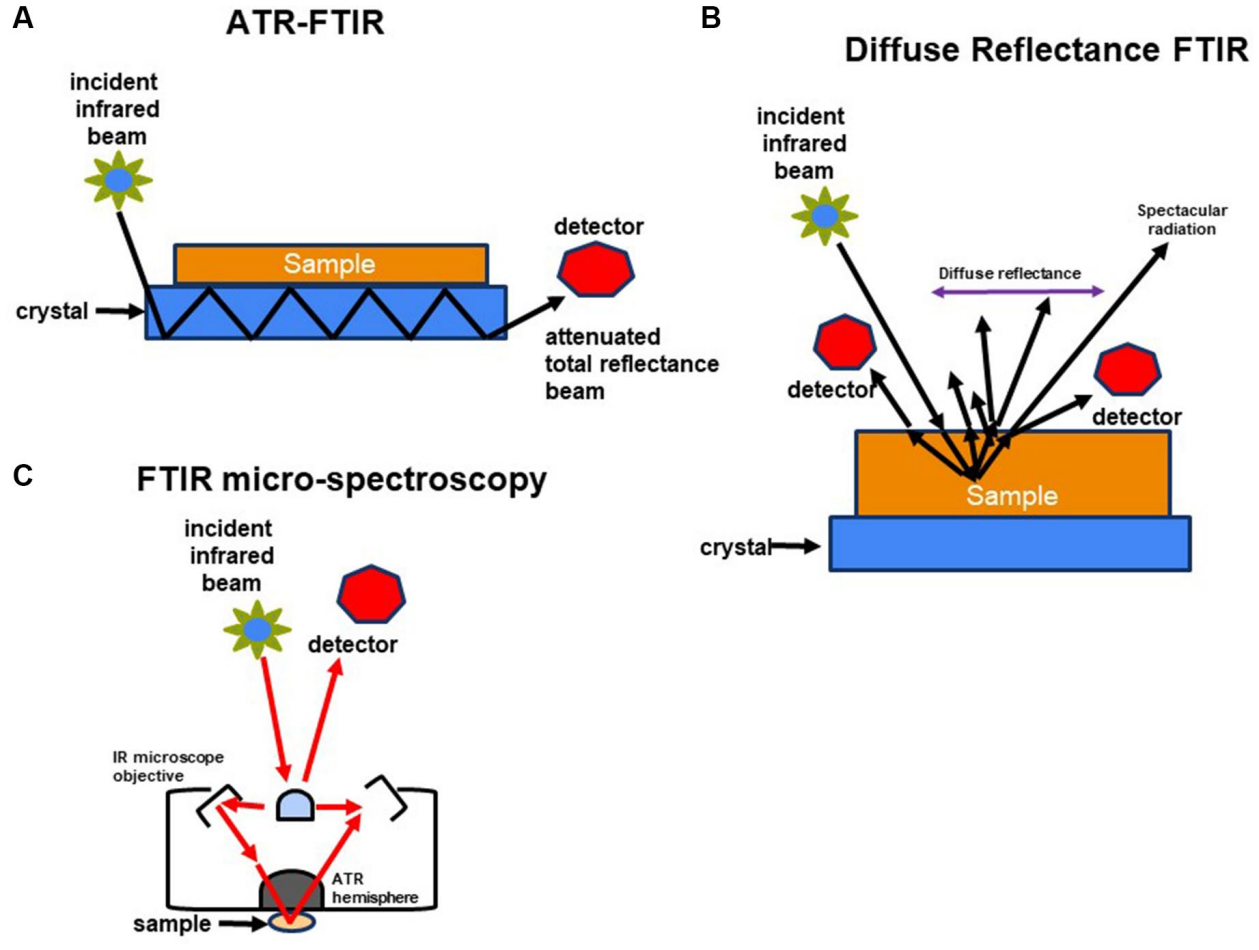
Schematic representation of methods used to characterize molecular composition of microorganisms: **(A)** FTIR-attenuated total reflectance, **(B)** diffuse reflectance FT-IR (DRIFT), and **(C)** FTIR-microspectroscopy.

In the case of ATR FT-IR, the sample is situated on a densely refractive crystal, usually of higher refractive index (Figure 2A). This method requires minimal or no sample preparation. The IR beam reflects off the crystal’s inner surface, giving rise to an evanescent wave that extends beyond the crystal’s surface and interacts with the sample in intimate contact with the ATR crystal (Baker et al., 2014; Bottum et al., 2023). The sample absorbs some of the evanescent wave’s energy, and the resulting reflected radiation reaches the IR spectrometer’s detector upon exiting the crystal. DRIFT involves directing the IR beam into the sample, where it is reflected, scattered, and transmitted through the sample material (Figure 2B). The IR light that becomes diffusely scattered within the sample and returns to the detector optics is termed diffuse reflection. Lastly, FT-IR micro-spectroscopy is an innovative technique that combines an FT-IR spectrometer with a microscope (Figure 2C). This approach enables the examination of limited areas on surfaces, such as agar plates, and facilitates the acquisition of reflectance or transmittance spectra from samples consisting of a few hundred cells, like microcolonies that develop within 6 to 10 h (Bhargava, 2012; Watanabe et al., 2023).

When subjecting a sample of a substance to continuous infrared (IR) light radiation, a distinctive resonance absorption band with intricate, fingerprint-like characteristics is obtained. The intensity of these absorption bands arises from scanning before and after the IR beam traverses the substance. Instead of isolated peaks, the frequencies and intensities of the IR bands exhibit distinctive, broad, and intricate profiles. These profiles serve as signatures that can be harnessed for the identification, characterization, and quantification of the sample. Upon radiating a sample containing bacterial cells, the resulting IR spectrum encapsulates the overall chemical composition of the sample. This spectrum holds the capability to provide insights into taxonomic variations due to inherent chemical distinctions or to identify chemical alterations resulting from exposure to challenging environments.

## 4. Applications of FT-IR in microbial cell biology

FT-IR spectroscopy has found numerous applications in the field of microbiology due to its ability to provide rapid and non-destructive information about the molecular composition of microorganisms. It is also used to investigate bacterial cell wall composition, studies biofilm formation and antibiotic resistance, monitor microbial growth and metabolic activity, and explore microbial community dynamics in complex environments. Furthermore, FT-IR spectroscopy provides insights into bacterial interactions with other microorganisms, host cells, and environmental factors, offering valuable information about microbial physiology and behavior. Thus, this technique finds widespread applications in microbiology, providing valuable insights into bacterial physiology, ecology, and interactions (Maquelin et al., 2000; Tiquia-Arashiro, 2019a; Jansson et al., 2023). Below reviews key applications of FT-IR in microbiology:

### 4.1. Microbial identification

FT-IR spectroscopy has been used for the identification of microorganisms based on their unique spectral fingerprints (Table 2). The FT-IR spectra of microbial cells, including bacteria, yeast, and fungi, contain characteristic peaks that reflect the presence of specific biomolecules (lipids, proteins, carbohydrates) and their functional groups. These spectral patterns can be compared to reference spectra in databases to identify the microorganisms. Microorganisms contain diverse biomolecules like proteins, lipids, nucleic acids, and carbohydrates, which exhibit characteristic absorption bands in the infrared spectrum. For bacterial identification, the FT-IR spectra of bacterial samples can be compared to spectral databases or reference spectra, researchers can identify bacterial species or strains based on their unique spectral features (Lin et al., 1998; Naumann et al., 2001; Dziuba et al., 2007; Rebuffo-Scheer et al., 2008; Novais et al., 2019; Feng et al., 2020; Brito and Lourenço, 2021). The ability of FT-IR spectroscopy to detect bacteria relies on the fact that the chemical composition and structure of bacterial biomolecules vary among different species and strains, resulting in distinct infrared absorption patterns. These patterns enable differences and identification. To enhance the accuracy and efficiency of bacterial detection and classification using FT-IR spectroscopy, researchers employ advanced statistical analysis methods like Principal Component Analysis (PCA) (Lasch et al., 2004; Bombalska et al., 2011), hierarchical clustering analysis (HCA) (Lasch et al., 2004; Dziuba et al., 2007; Bombalska et al., 2011; Feng et al., 2020), linear discriminant analysis (LDA) (Brito and Lourenço, 2021), stepwise discriminant analysis (SDA) (Lasch et al., 2004), and Ward’s algorithm (Dziuba et al., 2007; Table 2). By developing spectral libraries or databases that encompass a wide range of bacterial species, robust and reliable models for bacterial identification can be established (Martak et al., 2019; Tessaro et al., 2023; Yang et al., 2023). In bacterial identification, most analyses of bacterial samples have been observed to fall within the mid-IR region (4,000 to 600 cm^−1^) mainly because absorption patterns of functional groups in biological molecules are observed in this particular region as sharp fundamental vibrations, rather than broad overtones or harmonics which are found in the near-IR (AlMasoud et al., 2021).

**Table 2:** Applications of FT-IR in microbial identification.

Research goal	Microorganism	Data analysis	Reference
Compare the macrosample and microsample methods to evaluate which approach is more suited to the identification of *Listeria* spp. based on FT-IR spectra.	*Listeria* spp.	Leave-one-out method	Rebuffo-Scheer et al. (2008)
Determine the suitability of FT-IR as a supplement to MALDI-TOF MS for the identification and typing and identification microorganisms	*Escherichia coli* and *Shigella* species.	Correlation analysis, Principal component analysis (PCA), and hierarchical clustering analysis (HCA).	Feng et al. (2020)
Develop and validate an FTIR-ATR method for rapid identification of contaminants in pharmaceutical products	*Bacillus subtilis, Candida albicans, Enterococcus faecium, Escherichia coli, Micrococcus luteus, Pseudomonas aeruginosa, Salmonella typhimurium, Staphylococcus aureus,* and *Staphylococcus epidermidis.*	PCA and linear discriminant analysis (LDA).	Brito and Lourenço (2021)
Evaluate the potential of FT-IR for rapid identification of *Bacillus* isolates	*Bacillus cereus, Bacillus mycoides, Bacillus thuringiensis,* and other *Bacillus*, and non-*Bacillus* species.	**-**	Lin et al. (1998)
Differentiate and identify different lactic and propionic acid bacteria using (artificial neural networks) ANNs and FT-IR analysis; Expand the library of FTIR spectra of microorganisms	*Lactobacillus, Lactococcus, Leuconostoc, Propionibacterium, Streptococcus,* and *Lactobacillus.*	Custer analysis, Pearson’s correlation coefficient and Ward’s algorithm	Dziuba et al. (2007)
Study isolates belonging to the species *Campylobacter coli* and *Campylobacter jejuni* and to compare FT-IR typing schemes with established genomic profiles based on enterobacterial repetitive intergenic consensus PCR (ERIC-PCR)	*Campylobacter coli* and *Campylobacter jejuni*	HCA, stepwise discriminant analysis (SDA)	Lasch et al. (2004)
Use FTIR spectroscopy for the detection of the spectral parameters representing biochemical differences between species of the bacteria and fungi as well as different physiological states of the bacteria, i.e., endospores and vegetative cells of Bacillus spp.	*Bacillus cereus, Bacillus atrophaeus, Bacillus megaterium, Bacillus subtilis, Escherichia coli, Micrococus luteus, Pantoea agglomerans, Alternaria alternata, Candida albicans, Cladosporium herbarum, Penicillium brevicompactum* and *Penicillium chrysogenum*	PCA, HCA	Bombalska et al. (2011)

One of the key challenges in the use of FT-IR for microbial identification is the complexity of microbial samples, leading to the potential for overlapping spectral signals and difficulties in accurate identification (Wenning and Scherer, 2013). Moreover, the lack of standardized protocols and extensive databases can impede consistent and reliable microbial identification, particularly for less studied or newly discovered microorganisms (Franco-Duarte et al., 2019; Feng et al., 2020). In certain cases, the resolution of infrared spectroscopy might not be adequate to distinguish between closely related microbial species or strains, limiting its efficacy in precise microbial identification (AlMasoud et al., 2021). Microbial species exhibit considerable diversity, and within each species, there can be substantial variability due to genetic, environmental, and phenotypic factors (Feng et al., 2020).

Capturing this diversity in an FT-IR library would necessitate the inclusion of a wide range of strains and conditions, requiring extensive sampling and data collection efforts. Ensuring the standardization of sample preparation, data acquisition, and analytical protocols across different laboratories is crucial for the reproducibility and comparability of spectral data. Variations in experimental procedures can lead to inconsistencies in spectral profiles, making it challenging to build a reliable and consistent FT-IR library. Moreover, analyzing and interpreting complex spectral data from diverse microbial species requires advanced computational tools and expertise. Developing robust algorithms and software for efficient data processing, feature extraction, and classification of microbial spectra is essential for effective library management and utilization. However, despite these limitations, infrared spectroscopy remains instrumental in the rapid and non-destructive identification of microorganisms, aiding in the timely diagnosis of infections and the implementation of appropriate treatment strategies. Furthermore, it contributes to environmental monitoring and various biotechnological applications, demonstrating its significant role in the field of microbial identification despite its shortcomings.

### 4.2. Bacterial typing

FT-IR can differentiate bacterial strains and classify them into different groups or species based on their spectral profiles. This aids in bacterial typing, strain characterization, and epidemiological studies (Goodacre et al., 2000; Perkins et al., 2005; Yu, 2005; Wang et al., 2019; Feng et al., 2020; AlMasoud et al., 2021; Table 3). Whole living cells can be analyzed non-destructively, which allows *in vivo* investigations. As an example, diffuse reflectance infrared spectroscopy (DRIFT) was used to discriminate among 36 strains of vegetative *Bacillus* cells and their spores (Goodacre et al., 2000; Table 3). Different serovars of *Salmonella enterica* have been discriminated by mid-FTIR in attenuated total reflection (ATR) mode applying soft independent modeling of class analogy (SIMCA modeling) (Perkins et al., 2005; Glassford et al., 2013). Discrimination of endospores by mid-FTIR in ATR mode followed by the application of PCA (Goodacre et al., 2000; Perkins et al., 2005; Yu, 2005; Wang et al., 2019; Feng et al., 2020), hierarchical cluster analysis (HCA) (Perkins et al., 2005; Wang et al., 2019; Feng et al., 2020), canonical variates analysis (CVA/DFC) (Goodacre et al., 2000), correlation analysis (Feng et al., 2020), and SIMCA (Perkins et al., 2005) remained possible even after autoclaving of the samples (Perkins et al., 2005; Table 3). Libraries have been developed to relate spectral absorbance peaks of key functional groups present in proteins, carbohydrates, lipids, or nucleic acids (Yu and Irudayaraj, 2005; Fadlelmoula et al., 2022). Spectra of biological samples can be divided into different regions or windows. The typical fingerprint region for microorganisms is between wavenumbers of 650 cm^−1^ and 1,800 cm^−1^ originating from cellular carbohydrate compounds and proteins.

**Table 3:** Applications of FT-IR in bacterial typing.

Research goal	Microorganism	Data analysis	Reference
Typing of 36 *Bacillus* spp. and rapid detection of bacterial spores	*Bacillus* spp.	Principal component analysis (PCA) and Canonical variates analysis (CVA/DFA)	Goodacre et al. (2000)
Classification of bacterial endospores	*Bacillus* and *Clostridium* spores.	PCA, hierarchical clustering analysis (HCA), and Soft independent method of class analogy (SIMCA).	Perkins et al. (2005)
Use FT-IR microspectroscopy to whole microbial cells establish a spectroscopic fingerprinting basis for effective discrimination of microbial cells	*Salmonella, Escherichia coli, Yersinia enterocolitis,* and *Shigella boydii.*	PCA and CVA	Yu (2005)
Application of synchrotron radiation-based FTIR (SR-FTIR) spectroscopy to discriminate 10 bacterial strains in combination with chemometric methods.	*Salmonella, Shigella, Rhodococcus, Listeria, Yersinia, Vibrio,* and *Staphylococcus* strains.	PCA and HCA	Wang et al. (2019)
Determine the suitability of FT-IR as a supplement to MALDI-TOF MS for the identification and typing and identification microorganisms.	*Escherichia coli* and *Shigella* species.	Correlation analysis, PCA, and HCA.	Feng et al. (2020)
Develop a fast and reproducible non-molecular method to differentiate pure samples of *Bacillus* spores originating from different species as well as to identify spores in a simple matrix, such as the clay mineral, bentonite.	*Bacillus atrophaeus, Bacillus brevis, Bacillus circulans, Bacillus lentus, Bacillus megaterium,* *Bacillus subtilis* and *B. thuringiensis*	PCA, HCA, and SIMCA	Brandes and Brandl (2011)

Discrimination of vegetative cells and spores of *Bacillus circulans* was possible using FT-IR and subsequent chemometrical analysis of the spectra (Fadlelmoula et al., 2022). By FT-IR spectroscopy, spores of *Bacillus thuringiensis*, *B. subtilis*, and *B. megaterium* were easily distinguished. In some cases, however, IR fingerprints obtained by chemometrical analysis of spores of *B. atrophaeus*, *B. brevis*, *B. circulans*, and *B. lentus* clustered close together making discrimination difficult. The distance trees resulting from HCA based on FT-IR investigations of pure cultures agreed to phylogenetic trees derived from classical molecular methods based on 16S rRNA gene sequences (Zhao et al., 2006). Brandes and Brandl (2011) showed that spores originating from different *Bacillus species* can be discriminated against by applying FT-IR and subsequent multi-scaling chemometrical data treatment (Brandes and Brandl, 2011). A study carried out by Wang et al. (2019) proved the ability of FT-IR to distinguish between 16 types of foodborne pathogenic bacterial strains and was supported by multivariate analysis such as PCA and HCA of FT-IR data. In this study the authors found that a specific spectral region from 1,300 to 1,000 cm^−1^ which corresponds to phosphate and polysaccharide vibrations was successfully employed to discriminate bacterial strains (Smith, 2011; Wang et al., 2019).

One of the primary limitations in bacterial typing is related to the need for high resolution to differentiate between closely related strains or species, which may not always be achievable with standard infrared spectroscopy (AlMasoud et al., 2021). Moreover, the technique might struggle with complex microbial samples, leading to overlapping signals and difficulties in accurately distinguishing between different bacterial types (Perkins et al., 2005; AlMasoud et al., 2021). To address this challenge of achieving high resolution and differentiating closely related bacterial strains or species in bacterial typing using infrared spectroscopy, the integration of advanced data processing techniques such as machine learning algorithms and multivariate analysis could be beneficial (AlMasoud et al., 2021). These methods can enhance the spectral analysis by enabling the identification of subtle differences and patterns within complex microbial samples, thereby improving the accuracy of bacterial classification. Leveraging these computational tools alongside infrared spectroscopy can provide a comprehensive and robust framework for precise bacterial typing, allowing for the differentiation of closely related strains with greater resolution and reliability.

### 4.3. Microbial growth phases monitoring

FT-IR can monitor changes in microbial growth phases by tracking alterations in the spectral profiles of cells during different growth stages (Portenier et al., 2005; AlQadiri et al., 2008; Corte et al., 2011; Grace et al., 2020; Kochan et al., 2020; Spain and Funk, 2022; Table 4). This helps in understanding microbial physiology and metabolism (Tiquia-Arashiro, 2014). In batch cultures, bacterial growth is usually accompanied by four distinct stages which are clearly visible on the growth curve: (1) lag, (2) exponential (log), (3) stationary and (4) death phase (Cooper, 1991; Tiquia et al., 2004, 2008; Rolfe et al., 2012; Wang et al., 2015; Kochan et al., 2020). Knowledge of the bacterial growth stage, bacterial numbers and growth kinetics is needed in research and commercial applications. The basis of the traditional methods to determine growth phase is cell numbers which can be established using standard plate counting or by optical density (Stuart, 2004; Tiquia, 2010a; Nguyen et al., 2013; Plecha et al., 2013; Patel et al., 2019). However, any biochemical changes that could reflect microbial physiology cannot be described by these methods (Stuart, 2004; Tiquia, 2010a; Oest et al., 2018).

**Table 4:** Applications of FT-IR in monitoring microbial growth phases.

Research goal	Microorganism	Data analysis	Reference
Detect biochemical changes that occur during bacterial growth phases in batch culture.	*Escherichia coli* and *Listeria innocua*	Soft independent method of class analogy (SIMCA) and Principal component analysis (PCA)	AlQadiri et al. (2008)
Study the changes in nordic microalgal strains in relation with the logarithmic and stationary growth phases.	*Chlorella vulgaris, Scenedesmus* sp.*, Haematococcus pluvialis* and *Coelastrella* sp.	**-**	Spain and Funk (2022)
Determine the susceptibility of bacterial cells to three endodontic medicaments during exponential growth, stationary phase and starvation phase	*Enterococcus faecalis*	**-**	Portenier et al. (2005)
Detect the chemical composition of bacteria within the same phase (intra-phase).	*Staphylococcus aureus*	Student’s *t*-test	Kochan et al. (2020)
Analyze the molecular transitions and lipid accumulation in freshwater green microalgal species during their growth phases.	*Monoraphidium contortum, Pseudomuriella* sp. and *Chlamydomonas* sp.	PCA	Grace et al. (2020)

FT-IR spectroscopy detected significant changes in the chemical composition of bacteria through its different growth stages (Stuart, 2004; Spain and Funk, 2022). For the lag and log phases, these changes were mainly related to the various relative amounts of nucleic acid, accompanied by changes in protein composition (Kochan et al., 2020; Semeraro et al., 2023). The dominant spectral differences were associated with relative nucleic acid content, which reached its highest level after 60 and 90 min (Stuart, 2004; Kochan et al., 2020). This was expressed by bands at 1,215, 1,085, and 965 cm^−1^ in ATR. Further to that, an alteration in the protein composition (Amide II/Amide I) and substantial changes in relative carbohydrate content, visible via bands at 1,035 cm^−1^ (ATR) were observed. These changes may reflect the cellular activity aimed at adaptation to a new environment or in preparation for division (Stuart, 2004; AlQadiri et al., 2008; Spain and Funk, 2022). The results demonstrate the possibilities offered by multimodal vibrational spectroscopies (e.g., ATR-FTIR) toward providing a biochemical characterization, enabling the study of microbial physiology even given low bacterial numbers. Such biochemical probing opens a new door toward studying lag-phase related events (Stuart, 2004; Kochan et al., 2020; Spain and Funk, 2022). So, FT-IR demonstrates changes in the overall chemical composition of bacterial populations during growth (Zeroual et al., 1994; Stuart, 2004; Corte et al., 2011; Kochan et al., 2020; Tata et al., 2023).

FT-IR spectroscopy has been proven essential in elucidating bacterial chemical changes and understanding microbial physiology during different growth stages. However, its limited ability to capture subtle cellular composition changes and challenges in data interpretation pose constraints for comprehensive monitoring of microbial growth phases. Spectral overlap and diverse biochemical compositions across microbial species further complicate the establishment of a universal standard for interpreting FT-IR spectra. Additionally, the technique’s incapacity to capture rapid changes in cellular composition restricts its effectiveness for real-time analysis of microbial growth in dynamic environments. To overcome the challenges associated with using FT-IR spectroscopy to monitor microbial growth phases, the integration of complementary techniques and advanced data analysis methods can enhance the depth and accuracy of the analysis. Coupling FT-IR with high-resolution microscopy and flow cytometry can provide additional spatial and temporal information, enabling a more comprehensive understanding of cellular changes during different growth stages. Leveraging multivariate data analysis approaches, such as PCA and partial least squares regression (PLSR), can facilitate the deconvolution of complex spectral data and enable the identification and quantification of individual biochemical constituents, addressing the challenge of spectral overlap and variability across microbial species. Furthermore, the development of standardized protocols and reference databases specific to different microbial growth phases can aid in the interpretation and comparison of FT-IR spectra, fostering a more consistent and reliable analysis framework. Integration of these strategies can enhance the utility of FT-IR spectroscopy in monitoring microbial growth phases, providing a more holistic and detailed understanding of microbial physiology and metabolism.

### 4.4. Characterization of microbial cell wall components

FT-IR has been used to analyze the composition of microbial cell walls (Table 5), to study changes in cell phenotype (Galichet et al., 2001), elucidate changes in functional groups among Gram-positive and Gram-negative bacteria (Jiang et al., 2004; Tang et al., 2013; Zhang et al., 2023), determine the interactions with nanoparticles with the cell wall (Nadtochenko et al., 2005; Huang et al., 2017), monitor drug interactions with bacteria cells (Zlotnikov et al., 2023), characterize the functional role of extracellular polysaccharides and lipopolysaccharide (LPS) extracted from endophytic *Pseudomonas putida* against rice blast (Ashajyothi et al., 2023), and compare structural components of the cell wall of different algal strains logarithmic and stationary growth phases (Spain and Funk, 2022; Table 5). Changes in these structural components (e.g., peptidoglycan, lipopolysaccharides) can indicate bacterial responses to various conditions.

**Table 5:** Applications of FT-IR in characterization of microbial cell wall components.

Research goal	Microorganism	Data analysis	Reference
Understand the relation of the functional groups on the cell wall with the changes observed in the chemical cell surface properties in aqueous electrolyte solutions at different pH values.	*Aquabacterium commune*	**-**	Ojeda et al. (2008)
Assess the cell wall compositions of nordic microalgal strains.	*Chlorella vulgaris, Scenedesmus* sp.*, Haematococcus pluvialis* and *Coelastrella* sp.	**-**	Spain and Funk (2022)
Determine the structural changes of the *Escherichia coli* cell membranes during TiO_2_ photocatalysis.	*Escherichia coli*	**-**	Nadtochenko et al. (2005)
Discriminate mycobacteria and Gram-negative bacteria by assessing specific characteristic spectral features.	*Mycobacterium* spp. *Escherichia coli* strains, and *Pseudomonas putida.*	Direct classical least squares (DCLS)	Tang et al. (2013)
Investigate the effects of α-glucosidase on the cell wall of yeast bacteria.	*Saccharomyces cerevisiae*	**-**	Galichet et al. (2001)
Obtain functional group specific information on bacterial surfaces in aqueous solutions and observe their variation as a function of cell structure (Gram-positive versus Gram-negative cells) and solution composition.	*Bacillus subtilis, Bacillus lichenifermis, Pseudomonas stutzeri* and *Pseudomonas aeruginosa*	-	Jiang et al. (2004)
Investigate the interaction between TiO_2_ nanoparticles and cell membrane	*Escherichia coli* K-12	-	Huang et al. (2017)
Determine if cell wall-associated polysaccharides of *Pseudomonas putida* could elicit defense against rice blast.	*Pseudomonas putida*	-	Ashajyothi et al. (2023)
Monitor the drugs interaction with *Escherichia coli* cells localized in macrophages for diagnosis and treatment control of respiratory diseases	*Escherichia coli* JM109	-	Zlotnikov et al. (2023)
Determine the impact of reactive oxygen species on cell activity and structural integrity of Gram-positive and Gram-negative bacteria	*Staphylococcus aureus* and *Escherichia coli*	-	Zhang et al. (2023)

One study utilized FT-IR and other spectroscopic methods to investigate the cell surface properties of *Aquabacterium commune* (Ojeda et al., 2008). The FT-IR signals revealed the presence of carbon, phosphorus, and nitrogen atoms in the bacterial cell wall, with specific signals demonstrating variations in response to changes in pH. These variations were linked to acid–base reactive carboxyl, phosphoryl, and amine functional groups, suggesting their involvement in the acid–base exchange reactivity observed during titration experiments. In a separate study, FT-IR spectroscopy was employed to analyze the isolated cell wall material of four algal strains (*Chlorella vulgaris*, *Coelastrella* sp., *Scendesmus* sp. B2-2, and *Haematococcus pluvialis*) during logarithmic or stationary growth phases (Spain and Funk, 2022). The spectral shape indicated typical carbohydrate (960–1,180 cm^−1^) and protein (amide II; 1,475–1,620 cm^−1^ and amide I; 1,620–1,710 cm^−1^) regions, while the lipid fraction (around 1,740 cm^−1^) showed minimal absorption, suggesting a low presence of fatty acids in the cell walls. For strain comparison, the FTIR spectra were normalized to the amide I band (indicating protein content) (Spain and Funk, 2022).

Another study (Huang et al., 2017) utilized two-dimensional Correlation Fourier Transformation Infrared spectroscopy (2D-FTIR-COS) to investigate the interaction between TiO_2_ nanoparticles and bacterial cell membranes using bacterial ghosts (BGs), which are non-living bacterial cell envelopes. The results suggested that the proteins in BGs exhibited a strong preference for interacting with TiO_2_ nanoparticles, while the interaction with characteristic functionalities in polysaccharides (C-OH) and phospholipids (P=O) was minimal. This observation was further confirmed by the settlement of TiO_2_ nanoparticles in the presence of specific biomolecules such as bovine serum albumin (BSA), alginate, and phosphatidylethanolamine (PE). The asynchronous map of 2D-FTIR-COS indicated a sequential bonding order of COO- > aromatic C=C stretching > NH, amide II > C=O, ketone, shedding light on the interaction between TiO_2_ nanoparticles and bacterial cell membranes in aquatic environments.

Zhang et al. (2023) determined the impact of reactive oxygen species on cell activity and structural integrity of Gram-positive and Gram-negative bacteria in electrochemical disinfection system. Around 70% of the cell wall mass is composed of -CH_3_- and -CH_2_-vibrational stretching bands, with changes in their positions suggesting alterations in the cell wall layers. Notably, *Staphylococcus aureus* (a Gram-positive bacterium) exhibited a pronounced blue shift trend in the -CH_2_ and -CH_3_ peaks, indicating increased fluidity in the cell wall bilayer, potentially induced by peroxidation. Conversely, *E. coli* (A Gram-negative bacterium) showed no significant dependency in peak positions despite disorder in cell wall components. Analysis within the 2,000–1,000 cm^−1^ region identified five prominent peaks, including amides, nucleic acids, lipid, and protein vibrations, PO_2_-profiles, and C–O–P symmetric stretches from oligo/polysaccharides. Changes in peak positions and intensities of amide and C–O–P suggested modifications in cell wall proteins and oligosaccharides. While the C–O–P band weakening in *E. coli* indicated potential damage due to electrochemical oxidation, *S. aureus* exhibited slight changes after 60 min. Damage to the C–O–P band, a vital component in lipopolysaccharides (LPS), could lead to LPS degradation and subsequent cell structure failure. The decrease in C–O–P intensity served as an indicator of cell damage, corroborated by inactivation efficiency results. Furthermore, changes in the PO_2_-band near 1,245 cm^−1^ suggested the disruption of the hydrogen phosphate group during the electrochemical oxidation process. However, the detection of specific C=O peaks representing peroxidation products might require extended treatment time for accurate identification.

Fourier-transform infrared (FT-IR) spectroscopy has been widely used to investigate microbial cell walls, yet its application faces notable limitations. Interpreting FT-IR data is complex and demands specialized expertise, posing a significant challenge. Sample preparation sensitivity can affect spectra quality, with issues like sample thickness and uniformity leading to potential distortions. Limited spatial resolution restricts the observation of microscopic cell wall variations. Overlapping peaks within spectra can complicate the identification of specific functional groups, potentially leading to misinterpretations. Dependence on reference spectra, particularly for scarce data, may hinder compound characterization. FT-IR spectroscopy’s sensitivity to environmental factors, such as temperature and humidity, can introduce variability in results. While offering insights into cell wall chemical composition, it may lack the resolution required for understanding intricate cell wall interactions, limiting its use in certain research contexts. Considering these limitations is crucial for ensuring accurate and meaningful interpretation of FT-IR results in cell wall analysis. Standardizing sample preparation protocols, including precise control of sample thickness, uniformity, and purity, can minimize variability and ensure the reproducibility of spectral results. Building comprehensive reference databases for microbial cell wall components can facilitate more accurate comparison and interpretation of FT-IR results. Implementing rigorous environmental controls during data acquisition, such as temperature and humidity regulation, can help mitigate variations in spectral data. Furthermore, combining FT-IR with other high-resolution structural analysis techniques, such as electron microscopy and atomic force microscopy, can provide a more comprehensive understanding of the complex interactions within microbial cell walls.

## 5. Use of FT-IR spectroscopy in environmental microbiology

The study of microbial ecology has undergone transformative advancements due to innovative analytical techniques. Among these, Fourier Transform-Infrared (FT-IR) spectroscopy has emerged as a pivotal tool, enabling researchers to delve into the world of microorganisms with unprecedented precision and depth. FT-IR spectroscopy holds the potential to unravel the complexities of microbial communities, shedding light on their composition, metabolic activities, and responses to environmental changes. It also offers insights into the molecular signatures that characterize microorganisms, paving the way for a comprehensive understanding of microbial ecosystems and their vital roles in various habitats. This exploration into the utilization of FT-IR spectroscopy in microbial ecology opens new avenues for deciphering the hidden dynamics that govern microbial interactions and their impact on broader ecological systems.

### 5.1. Monitoring microbial biofilms

Microbial biofilms, complex communities of microorganisms adhering to surfaces and encased in self-produced extracellular polymeric substances (EPS), have garnered significant attention due to their diverse roles and impact on various fields, including medicine, industry, and environmental science (Schmitt et al., 1995; Schmitt and Flemming, 1998; Bosch et al., 2006; Mukherjee et al., 2011; Quilès et al., 2016; Tugarova et al., 2017; Di Martino, 2018; Miao et al., 2019; Gieroba et al., 2020; Cheah and Chan, 2022).

The use of Fourier Transform-Infrared (FT-IR) spectroscopy has proven to be a valuable and versatile approach in monitoring bacterial (Bosch et al., 2006; Elzinga et al., 2012; Quilès et al., 2016; Tugarova et al., 2017; Singhalage et al., 2018; Cheeseman et al., 2021; Kamnev et al., 2023), fungal (Singhalage et al., 2018; Cheeseman et al., 2021; Dimopoulou et al., 2021), algal (Tong and Derek, 2021; Wang et al., 2021) and microbial community (Schmitt and Flemming, 1998; Oberbeckmann et al., 2014; Gong et al., 2019; Miao et al., 2019) biofilms (Table 6). As an analytical tool, the attenuated total reflection (ATR) offers a further possibility to directly investigate the chemical composition of smooth surfaces of various materials (Smith, 1998; Elzinga et al., 2012; Cheeseman et al., 2021; Tong and Derek, 2021; Watanabe et al., 2023). This can be achieved practically without any sample preparation. With respect to biofilm research, this offers the significant advantage that the sample can be investigated in a relatively undisturbed state. Especially for membrane and polymer investigations, ATR is a helpful analytical tool. A further advantage of FTIR-ATR-spectroscopy in biofilm research is the possibility of measuring in aqueous media as well as investigating the development of a biofilm *in situ*, non-destructively and in real time directly at the substratum/liquid interface (Schmitt et al., 1995; Schmitt and Flemming, 1998; Oberbeckmann et al., 2014; Miao et al., 2019; Jansson et al., 2023). The major advantage of the ATR method is that the biofilm can be observed non-destructively, directly, on-line and in real time (Schmitt and Flemming, 1998; Tiquia-Arashiro, 2012). FT-IR can provide useful information on the functional groups of EPS that play an adhesive and cohesive role in maintaining biofilms (Geoghegan et al., 2008; Di Martino, 2018). The EPS matrix plays a pivotal role in biofilm formation, stability, and protection. FT-IR spectroscopy assists in elucidating the composition of EPS, which includes polysaccharides, proteins, and other biomolecules. By studying changes in specific bands related to carbohydrate and protein vibrations, researchers can assess the dynamic nature of EPS as biofilms develop, mature, and respond to environmental cues (Bosch et al., 2006; Ojeda et al., 2008; Mukherjee et al., 2011; Bowman et al., 2018; Cheeseman et al., 2021; Cheah and Chan, 2022).

**Table 6:** Applications of FT-IR in monitoring microbial biofilms.

Research goal	Microorganism	Data analysis	Reference
Determine the effect of pH and contact time on the attachment of bacteria to a surface of hematite in the process of biofilm formation	*Shewanella putrefaciens*	**-**	Elzinga et al. (2012)
Study the characteristics of a bacterial biofilm.	*Bordetella pertussis*	Analysis of variance (ANOVA) and Fisher’s least significant difference (LSD)	Bosch et al. (2006)
Characterize biofilm formation on the surface of ZnSe discs by a rhizobacterium.	*Azospirillum brasilense*	**-**	Tugarova et al. (2017)
Analyze the effect of antimicrobial peptides on nascent bacterial biofilm.	*Pseudomonas fluorescens*	Sneddon model	Quilès et al. (2016)
Use FTIR spectroscopic analyses to study biofilms formed on a solid surface or liquid/air interface along with the macromolecular biofilm matrix and its main macrocomponents,	*Azospirillum baldaniorum*	**-**	Kamnev et al. (2023)
To confirm the value of FT-IR spectroscopy in biology, medicine, and pharmacy as effective tools for bacterial product characterization.	*Streptococcus* spp.	-	Gieroba et al. (2020)
Investigate the photosynthetic parameters, microstructures, biomass accumulation, and CO_2_ fixation rate of *Chlorella* sp. biofilms cultured under a series of light intensities	*Chlorella* sp.	-	Wang et al. (2021)
Study the relationships between the algal attachment rate onto membranes via biofilm and the compositional changes of the extracellular polymeric substances in the biofilm	*Amphora coffeaeformis, Cylindrotheca fusiformis* and *Navicula incerta*	ANOVA	Tong and Derek (2021)
Show differences between biofilms formed by Gram-positive methicillin-resistant *Staphylococcus aureus* (MRSA), Gram-negative *Pseudomonas aeruginosa*, and the yeast-type *Candida albicans* using synchrotron macro attenuated total reflectance-Fourier transform infrared (ATR-FTIR) microspectroscopy.	*Staphylococcus aureus, Pseudomonas aeruginosa,* and *Candida albicans*	Hierarchical cluster analysis (HCA), Principal component analysis (PCA)	Cheeseman et al. (2021)
Determine the adhesion capacity of eleven strains of the species, compare the metabolic fingerprint of the attached vs. planktonic cells, reveal the compounds implicated in the biofilm formation associated with wine spoilage.	*Brettanomyces bruxellensis*	PCA	Dimopoulou et al. (2021)
Examine the structural attributes of fungal-bacterial biofilms in comparison to bacterial (BBs) and fungal (FBs) biofilms.	*Enterobacter* sp. and *Aspergillus* sp.	-	Singhalage et al. (2018)
Investigate the development and properties of biofilm.	Microbial communities	-	Schmitt and Flemming (1998)
Investigate the biofilm formation by microorganisms originating from lake water on low-density polyethylene	Microbial communities	ANOVA	Gong et al. (2019)
Understand the structural and taxonomical variation of microbial biofilm communities on plastic fragments in coastal and offshore Northern European waters, with respect to season, geographical location and plastic type.	Microbial communities	-	Oberbeckmann et al. (2014)

The complex composition of biofilms can lead to challenges in identifying specific components due to overlapping spectral bands and the heterogeneous mixture of biomolecules and extracellular polymeric substances (EPS). Advanced spectral deconvolution techniques are necessary for accurate analysis. The inherent heterogeneity of biofilm structures introduces variability in FT-IR signals, making it difficult to establish consistent baseline data for comparison and identify subtle changes during biofilm development and responses to environmental stimuli. Environmental influences on biofilm formation and EPS composition, such as temperature and nutrient availability, require careful consideration and control during experimental design and data interpretation. Background signals and contaminants can interfere with the identification of specific biomolecules and functional groups within the biofilm, affecting the reliability of FT-IR analysis. The limited spatial resolution of traditional FT-IR spectroscopy may hinder precise characterization of localized changes within the biofilm matrix, emphasizing the need for complementary imaging techniques and high-resolution FT-IR imaging systems to enable more accurate spatial analysis of microbial biofilms. Employing advanced data processing and statistical modeling approaches, such as multivariate analysis and machine learning algorithms, can aid in interpreting complex FT-IR spectra and discerning subtle changes in biofilm development and response to environmental stimuli. Integration of complementary imaging techniques, such as confocal microscopy and atomic force microscopy, can provide spatially resolved information, complementing FT-IR spectroscopy and enabling more comprehensive characterization of localized biofilm structures and molecular distributions. Furthermore, the development of high-resolution FT-IR imaging systems and the incorporation of advanced data visualization tools can enhance spatial resolution and facilitate detailed mapping of biofilm composition and dynamics at the microscale level (Kamnev et al., 2023).

### 5.2. Functional profiling of microbial communities

Microbial communities within natural environments carry out a plethora of functions essential for nutrient cycling, organic matter decomposition, and ecosystem stability (Tiquia et al., 2006a,b; Tiquia, 2008, 2010b; Igisu et al., 2012; Mckindles and Tiquia-Arashiro, 2012; Flood et al., 2015; Tiquia-Arashiro and Grube, 2019; Duncan and Petrou, 2022; Naylor et al., 2022). The metabolic activities and functional profiles of the microbial communities are influenced by environmental factors, including temperature fluctuations, nutrient availability, and pollutant exposure (Liu et al., 2003; Wu et al., 2005; Gentile et al., 2006; Tiquia, 2010a, 2011; Dong et al., 2017; Qin et al., 2018; Tiquia-Arashiro, 2019b; Huang et al., 2020; Cabugao et al., 2022; Duncan and Petrou, 2022).

FT-IR spectroscopy has been crucial in comprehending the functional characteristics of microbial communities (Table 7). This method is commonly combined with genetic techniques like metagenomics and qPCR (Edwards et al., 2014; Lin et al., 2014; Andrei et al., 2015; Miao et al., 2019; Wang et al., 2021; Wilson et al., 2021 and Zada et al., 2021). Additionally, it is utilized alongside genetic fingerprinting techniques (Edwards et al., 2014), radioisotope labeling (Andrei et al., 2015; Wang et al., 2021; Wilson et al., 2021; Duncan and Petrou, 2022), as well as biochemical assays like community level physiological profiles (CLPP) (Artz et al., 2006) and enzyme assays (Miao et al., 2019). Other supporting analytical and imaging techniques include excitation emission matrix spectroscopy (EEMS) (Wilson et al., 2021) and scanning electron microscopy (SEM) (Miao et al., 2019).

**Table 7:** Applications of FT-IR in functional profiling of microbial communities.

Research goal	Microorganism	Data analysis	Reference
Investigate the genetic and functional diversity of bacteria and archaea in typical limestone (Kashmir Cave) and silicate-containing (Tiser Cave) caves.	Microbial communities	Nonmetric multidimensional scaling (NMDS)	Zada et al. (2021)
Evaluate community level physiological profiles of the microbial community in peat horizons of differing degrees of humification	Microbial communities	Principal components analysis (PCA), subsequent canonical variate analysis (CVA)	Artz et al. (2006)
Quantitatively link the abundance and distribution of specific microbial groups to the dynamics of specific plant-derived organic carbon compounds and environmental parameters at the ecosystem scale in a northern peatland	Microbial communities	paired *t* tests, chi-square test, distance-based linear model combined with a forward model selection method	Lin et al. (2014)
Determine the plasticity and taxonomic diversity of sea ice microalgae macromolecular composition, with a focus on how different environmental conditions influence macromolecular production and partitioning within cells and communities.	Microbial communities	Permutational multivariate ANOVA	Duncan and Petrou (2022)
Examine bacterial community structure and biochemical properties of organic matter by metabolite fingerprinting using FT-IR spectroscopy.	Microbial communities	Canonical correspondence analysis (CCA), Permutational multivariate ANOVA	Edwards et al. (2014)
Compare the prokaryotic assemblages from Ursu and Fara Fund hypersaline meromictic lakes (Transylvanian Basin, Romania) in relation to their limnological factors and infer their role in elemental cycling by matching taxa to known taxon-specific biogeochemical functions	Microbial communities	Correlation analysis, Permutational multivariate ANOVA	Andrei et al. (2015)
Probe the extent of environmental perturbations using high-resolution geochemical and microbial gene-based community profiling of anaerobically incubated material from three wetland habitats across a permafrost peatland	Microbial communities	Permutational multivariate ANOVA	Wilson et al. (2021)
Investigate the effects of copper oxide (CuO) NPs on freshwater sediment biofilms in terms of the functional properties and microbial community structure	Microbial communities	Linear discriminant analysis (LDA), Permutational multivariate ANOVA	Miao et al. (2019)
Stduy the transformation patterns of different S cycling microbial species in mangrove sediments amended with different microplastics and their associated microbial communities were investigated	Microbial communities	ANOVA	Wang et al. (2021)

**Figure 3:**
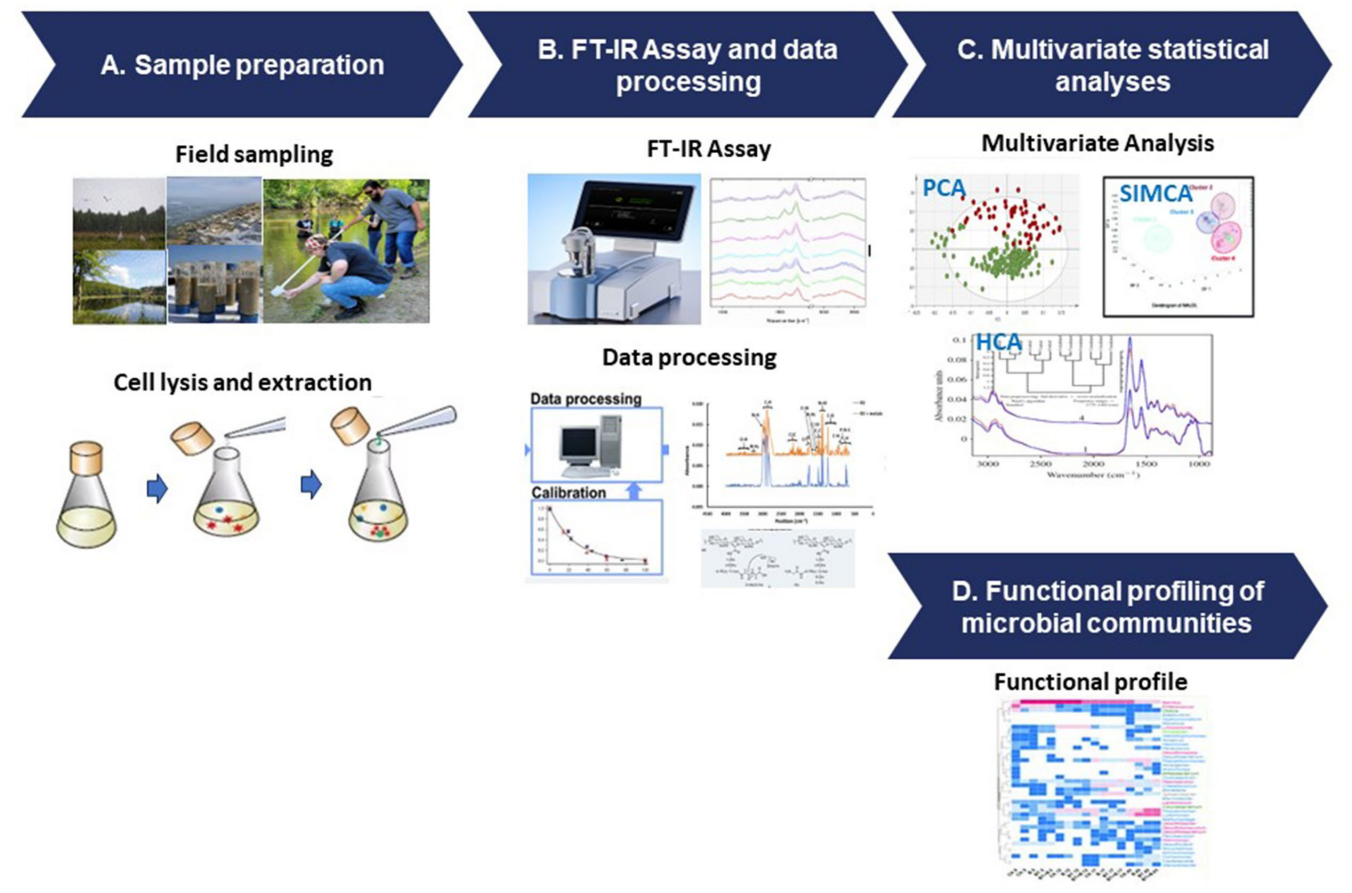
FT-IR protocol for microbial functional analysis. **(A)** Sample preparation, **(B)** FT-IR assay and data processing, **(C)** multivariate statistical analyses, and **(D)** functional profiling of the microbial communities.

The FT-IR protocol follows a structured process that involves sample preparation, data acquisition, and advanced data analysis techniques (Figure 3). To ensure accurate representation of microbial components, diverse samples are collected from various environments and undergo preparation steps such as cell lysis and extraction (Figure 3A). FT-IR spectroscopy generates unique spectral signatures revealing the biomolecular composition of the microbial samples, providing insights into lipids, proteins, carbohydrates, and nucleic acids (Figure 3B). The data analysis stage utilizes sophisticated tools like PCA and HCA and SIMCA (Figure 3C), enabling comparisons with reference databases to identify microbial taxa and their functional attributes (Figure 3D).

While FT-IR spectroscopy has proven to be a valuable tool for studying microbial functions in natural environments, it has encountered challenges in interpreting the complex data it generates. The acquired spectra demand a profound grasp of both spectroscopy and microbiology. To ensure meaningful comparisons and reliable findings across studies, standardized protocols and data analysis methods are imperative, given the diversity and variability in natural environments and microbial communities. Uniform protocols and robust analytical frameworks are essential for maintaining consistency and coherence in result interpretation, thereby bolstering the credibility and reproducibility of findings. Despite challenges, FT-IR spectroscopy serves as a bridge between molecular insights and ecosystem processes. By utilizing multivariate statistical tools such as PCA and cluster analysis, researchers can identify functional patterns and understand the roles of microorganisms in environmental dynamics. The integration of FTIR spectroscopy with other advanced techniques, including genetic analysis and stable isotope probing (Wang et al., 2023), holds promise for deeper insights into microbial functions, but requires careful consideration and methodological refinement to effectively merge these approaches.

### 5.3. Microbial stress and viability

The use of FT-IR spectroscopy for microbial stress and viability has emerged as a valuable approach for understanding the responses of microorganisms to various stressors (Table 8). Microorganisms encounter a multitude of stressors, including changes in nutrient availability, temperature fluctuations, pH variations, exposure to toxins, and other environmental challenges. Traditional methods for assessing microbial stress and viability often involve time-consuming culturing procedures and may not accurately represent the real-time physiological state of microorganisms (Kumar and Ghosh, 2019). FT-IR spectroscopy offers a rapid and comprehensive alternative for studying these aspects. When microorganisms undergo stress or changes in their physiological state, alterations occur in their biochemical composition, resulting in shifts in FT-IR spectra. Researchers can analyze these changes to assess the stress levels and viability of microorganisms (Moen et al., 2005, 2009; Alvarez-Ordóñez et al., 2011; Câmara et al., 2020). Monitoring these shifts allows researchers to infer stress-induced modifications within cellular components, including protein denaturation, lipid peroxidation, and changes in carbohydrate content, offering insights into microbial adaptation mechanisms (Moen et al., 2005, 2009; Alvarez-Ordóñez et al., 2011; Câmara et al., 2020). Employing various modes of FT-IR spectroscopy, including transmission, DRIFT, and ATR, researchers have examined changes in the composition and structure of whole bacterial cells in response to specific stress factors and plant-derived signals (Kamnev, 2008; Meyvisch et al., 2022). Investigations using FT-IR spectroscopy have revealed distinctive metabolic variations between epiphytic and endophytic strains of the *Azospirillum brasilense* species under conditions of heavy-metal stress (Kamnev, 2008). Furthermore, FT-IR spectroscopy has been utilized to study the metabolomic stress responses triggered by N-alkylpyridinium bromide surfactants in yeast strains *Saccharomyces cerevisiae* and *Candida albicans*, demonstrating its utility in probing stress-induced metabolic alterations in various microorganisms (Corte et al., 2014). Studies involving FT-IR spectroscopy have highlighted its sensitivity in detecting stressful conditions or pathological states, ranging from bacterial reactions to antibiotics, responses to starvation and environmental stressors, to encounters with pollutants and gene mutations, underscoring its potential in diverse research contexts (Melin et al., 2001; Kamnev et al., 2005; Portenier et al., 2005; Stehfest et al., 2005; El-Bialy et al., 2019; Câmara et al., 2020; Ribeiro da Cunha et al., 2020; Scarsini et al., 2021; Soares et al., 2022; Table 8).

**Table 8:** Applications of FT-IR in microbial stress and viability assessment.

Research goal	Microorganism	Data analysis	Reference
Understand stress responses triggered by antibiotic exposure	*Escherichia coli*	Savitzky–Golay (SG) and Loopy Multiplicative Scatter Correction (LMSC), Partial Least Squares Discriminant Analysis (PLS-DA) predictive models.	Ribeiro da Cunha et al. (2020)
Modulating the synergistic response of antibiotics to circumvent the mechanisms of bacterial resistance	*Staphylococcus aureus*	Hierarchical Cluster Analysis (HCA)	Soares et al. (2022)
Confirm the metabolic plasticity of microalgae in response to environmental stress (nutrient stress)	*Phaeodactylum tricornutum*	Standard Normal Variate (SNV), ANOVA	Scarsini et al. (2021)
Understand the modifications of single-cells biophysical profiles during different dehydration conditions using synchrotron radiation-based Fourier-transform infrared (S-FTIR)	*Lachancea thermotolerans*	Principal component analysis (PCA)	Câmara et al. (2020)
Understand the effect of different environmental stresses including heavy metal, nutritional, plant stress-response protein stresses on soil bacteria	*Azospirillum* sp.	**-**	Kamnev (2008)
Assess the effect of N-tetradecyltropinium bromide surfactant and some of its variants on yeast bacteria and human pathogens.	*Saccharomyces cerevisiae* and *Candida albicans*	QUANT2 algorithm	Corte et al. (2014)
Investigate the susceptibility of bacterial cells to three endodontic medicaments	*Enterococcus faecalis*	Student’s *t*-test	Portenier et al. (2005)
Determine the effect of nutrient stress on bacterial composition of phytoplankton algae	*Microcystis aeruginosa, Croococcus minutus, Nostoc* sp.*, Cyclotella meneghiniana,* and *Phaeodactylum tricornutum*	**-**	Stehfest et al. (2005)
Characterize the effect of γ-irradiation on bacteria.	*Deinococcus radiodurans*	Savitsky–Golay algorithm and Ward’s algorithm	Melin et al. (2001)
Develop FTIR micro-spectroscopy device for probing live bacteria and to compare the biochemical data obtained at the single-cell level with data obtained from clusters of thousands of cells	*Lactobacillus delbrueckii* subsp*. bulgaricus*	Savitzky–Golay algorithm, PCA	Meneghel et al. (2020)
Apply specific collection techniques and spectroscopy to differentiate between live and dead *Escherichia coli* O157:H7 cells, as well as cells subjected to various inactivation treatments, including heat, salt, UV, antibiotics and alcohol.	*Escherichia coli* O157:H7	Canonical variate analysis (CVA), Mahalanobis distances (MD)	Davis et al. (2012)
Determine if FT-IR could detect and differentiate between *Salmonella typhimurium* and *Salmonella enteritidis* live serotypes and between live and dead Salmonella cells.	*Salmonella typhimurium* MH 68123 and *Salmonella enteritidis* MH 42841	PCA, Mahalanobis distances, Soft independent modeling by class analogy (SIMCA)	Sundaram et al. (2012)

Microbial viability assessment is central to understanding the functional status of microbial populations in diverse environments (Braissant et al., 2020). It provides critical information about the metabolic activity, reproductive potential, and overall health of microorganisms (Nguyen et al., 2013; Pomaranski and Tiquia-Arashiro, 2016; Braissant et al., 2020). In microbial ecology, assessing viability aids in deciphering community responses to environmental fluctuations, resource availability, and biotic interactions (Tiquia et al., 2002; Cho et al., 2012; Berninger et al., 2018). Additionally, viability assessments are pivotal for monitoring the efficacy of antimicrobial treatments, studying microbial survival strategies, and predicting ecosystem responses to perturbations.

**Figure 4:**
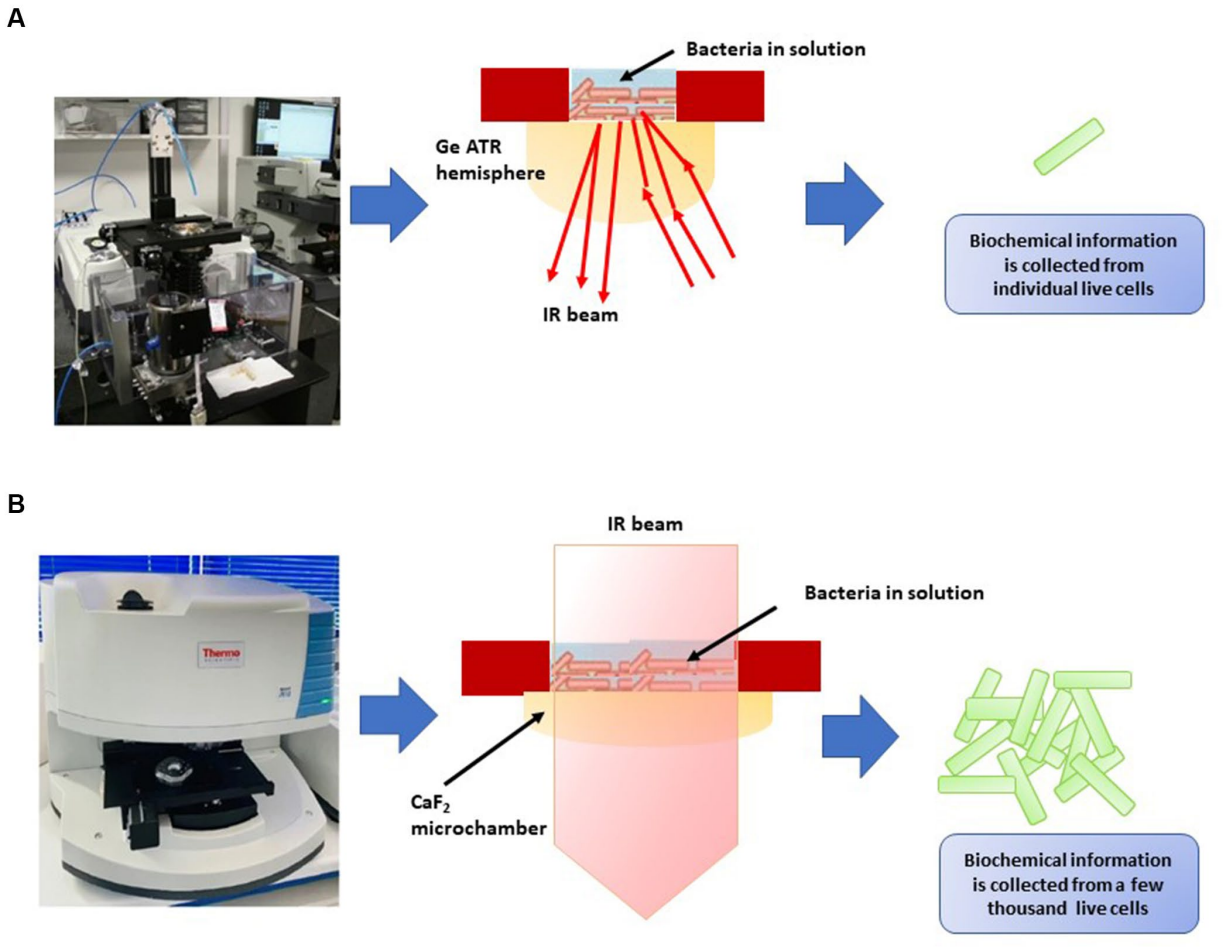
Two types of FT-IR developed by Meneghel et al. (2020). **(A)** Attenuated total reflectance-Fourier transform infrared (ATR-FTIR) inverted microscope for the analysis of individual bacterial cells in an aqueous environment using synchrotron radiation. An enlargement diagram of the liquid sampling area showing the ATR principle. **(B)** Thermal source-powered FTIR microscope for probing clusters of a few thousands of live cells in the mid-IR region (4,000–975 cm^−1^). Diagram shows the demountable liquid micro-chamber.

FT-IR spectroscopy has practical applications in discerning viable microbial cells and distinguishing between viable and non-viable ones (Davis et al., 2012; Sundaram et al., 2012; Meneghel et al., 2020; Table 8). Meneghel et al. (2020) developed two distinct FT-IR systems for analyzing the infrared spectra of bacteria in water, operating at different spatial resolutions (Figure 4). The first system integrated a custom-built attenuated total reflection inverted microscope with a synchrotron-based FT-IR spectrometer (Figure 4A), enabling the acquisition of spectra at the individual-cell level within the 1,800–1,300 cm^−1^ range. The second system utilized a transmission FT-IR microscope with a specially designed sample holder for liquid samples (Figure 4B), allowing the examination of viable cells across the entire mid-IR region by regulating the optical path length. These approaches helped identify potential cellular markers of bacterial populations, including the secondary structure of proteins, particularly the proportions of α-helix and β-sheet structures, as well as cell envelope components, specifically polar head groups of membrane phospholipids and complex sugars of the peptidoglycan cell wall. These components, already utilized for microbial identification, were also implicated in cryo-resistance mechanisms. Multivariate analysis of the spectra revealed that cryo-sensitive cells exhibited the highest cell heterogeneity and the highest content of proteins with the α-helix structure. Moreover, cluster analysis of bacterial cells highlighted phosphate and peptidoglycan vibrational bands associated with the cell envelope as potential markers of resistance to environmental conditions (Meneghel et al., 2020).

Sundaram et al. (2012) conducted research indicating the effectiveness of FT-IR in distinguishing between live and deceased cells of *Salmonella typhimurium* and *Salmonella enteritidis*. They discovered significant spectral variations in components such as the cell wall, cell membrane, cytoplasm, polysaccharides, proteins, peptides, amide bands, and nucleic acids. The Mahalanobis distance and SIMCA analysis yielded a perfect 100% accuracy in classifying live and dead cells of both *Salmonella* serotypes. However, the authors emphasized the need for further investigations to develop a comprehensive spectral library for different *Salmonella* serotypes and validate the method’s sensitivity and selectivity using standard microbiological procedures. Davis et al. (2012) also utilized FT-IR spectroscopy to differentiate live and dead *Escherichia coli* O157:H7 cells and those treated with various inactivation methods (heat, salt, UV, antibiotics, and alcohol). The study demonstrated the efficacy of partial least squares analysis and canonical variate analysis (CVA) in rapidly distinguishing between live and dead cells. This approach shows promise in reducing detection time compared to other techniques like fluorescent microscopy and EMA-qPCR, which necessitate lengthier preparation and detection times. The findings suggest potential applications of this FT-IR approach in analyzing food samples subjected to different inactivation treatments.

In these studies, changes in spectral features, such as alterations in protein and lipid content, served as indicators of cellular viability. By comparing FT-IR spectra of viable and non-viable cells, researchers can develop spectral fingerprints that aid in rapid and non-destructive viability assessment. The viability of microbial cells influences their biochemical makeup, leading to variations in FT-IR spectra. Viable cells generally exhibit well-defined spectral bands associated with intact proteins, lipids, nucleic acids, and carbohydrates. In contrast, non-viable cells or cells undergoing stress may exhibit shifts or reductions in these bands due to protein denaturation, lipid degradation, and changes in cellular structure (Davis et al., 2012; Sundaram et al., 2012; Meneghel et al., 2020). By analyzing these spectral changes, researchers can establish viability indicators that aid in distinguishing between live and non-live cells. While FT-IR spectroscopy holds promise for microbial viability assessment, challenges remain. The interpretation of complex spectral data requires advanced analytical techniques and expertise. Standardization of protocols and data analysis methods is essential for ensuring consistent results across studies. As technology advances, integrating FT-IR spectroscopy with other techniques, such as microscopy and flow cytometry, could provide a multi-faceted view of microbial viability and functional responses in ecological contexts.

### 5.4. Microbial biosorption studies with metals

Microbial biosorption has gained prominence as an environmentally friendly method for extracting heavy metals from water (Bowman et al., 2018; Tiquia-Arashiro, 2018, Pagnucco et al., 2023). This process involves a variety of interactions between microbial cell surfaces and metal ions, including physical adsorption, ion exchange, complexation, and surface precipitation (Tiquia-Arashiro, 2018; Tiquia-Arashiro and Pant, 2019; Pagnucco et al., 2023). FT-IR spectroscopy allows researchers to investigate the molecular-level alterations on microbial cell surfaces during metal binding. By examining the vibrational modes of functional groups like hydroxyl, carboxyl, and amino groups, FT-IR spectra offer insights into the chemical bonds formed between microorganisms and metal ions. The analysis of FT-IR spectra post-biosorption reveals shifts in peak positions or intensity, indicating changes in functional group vibrations. These modifications provide valuable information about the specific functional groups responsible for metal absorption and the ensuing chemical reactions on the microbial cell surface. Comprehensive analysis of biosorption of metal ions using FT-IR have been studied in bacteria (Pardo et al., 2003; Oh et al., 2009; Gutiérrez-Corona et al., 2016; Dhanwal et al., 2018; Saranya et al., 2018; Pagnucco et al., 2023), algae (Michalak et al., 2013; Gu and Lan, 2021) and fungi (George et al., 2012; Gutiérrez-Corona et al., 2016; Sundararaju et al., 2022; Table 9).

**Table 9:** Applications of FT-IR in microbial biosorption studies with metals.

Research goal	Microorganism	Data analysis	Reference
Study the biosorption process of Cr^3+^, Mn^2+^ and Mg^2+^ ions by a freshwater macroalga	*Cladophora glomerata*	**-**	Michalak et al. (2013)
Unravel the interactions of bacteria with Cr^6+^	*Streptomyces werraensis* and *Aspergillus niger*	**-**	Gutiérrez-Corona et al. (2016)
Assess the biosorptive capacity of Pb^2+^, Cd^2+^ and Cu^2+^ by bacteria.	*Pseudomonas stutzeri*	Langmuir and Freundlich models.	Oh et al. (2009)
Determine the biosorption of Cd^2+^, Cu^2+^, Pb ^2+^ and Zn ^2+^ by bacteria.	*Pseudomonas putida*	Langmuir and Freundlich models.	Pardo et al. (2003)
Study the biosorption of Cd^2+^, Cr^6+^, Cu^2+^, and Zn^2+^ by coral associated phosphate solubilizing bacteria.	*Cronobacter muytjensii*	Langmuir model.	Saranya et al. (2018)
Investigate the removal of Pb^2+^ and Hg^2+^ from aqueous solutions by bacteria isolated from estuarine sediments	*Aspergillus niger*	**-**	George et al. (2012)
Assess the biosorptive capacity of bacterial strains single and multi-metal solutions	*Klebsiella* spp. *Serratia* sp. and *Raoultella* sp.	**-**	Pagnucco et al. (2023)
Screened biosorption potential of bacteria isolated from soil contaminated with electroplating industrial effluents to multi-metals (Cu^2+^, Ni^2+^, Pb^2+^, and Cr6^+^)	*Bacillus cereus*	**-**	Dhanwal et al. (2018)
Evaluate the biomass of *Penicillium* sp. MRF1 as biosorbent for the removal of Ni^2+^ ions from electroplating industrial effluent.	*Penicillium* sp. MRF1	Analysis of variance (ANOVA); Freundlich isotherm model	Sundararaju et al. (2022)
Understand the functional groups at cell surface, and the interactions between metal ions (Cu^2+^, Zn^2+^, Pb^2+^, Hg^2+^, and Cd^2+^) and biosorbents	*Neochloris oleoabundans*	Langmuir model	Gu and Lan (2021)
Determine the physiological characteristics of *Spirulina* strains, and their chromium (VI) adsorption capacities	*Spirulina* spp.	Principal components analysis (PCA)	Meng et al. (2023)

In Michalak et al.’s (2013) work, they investigated the biosorption of Cr^3+^, Mn^2+^, and Mg^2+^ by the freshwater macroalga *Cladophora glomerata*, using FT-IR spectroscopy. The FTIR analysis identified key functional groups involved in the process, primarily carboxyl and hydroxyl. In Gutiérrez-Corona et al.'s (2016) review, FT-IR spectroscopy was employed to examine the cell walls of microorganisms like *Streptomyces werraensis* LD22 and *Aspergillus niger*, highlighting the specific functional groups involved in binding with chromium (Cr^6+^). The analysis revealed the participation of amine, hydroxyl, and carboxyl groups in Cr^6+^ binding within the cell walls of *S. werraensis* LD22, while in *Aspergillus niger*, hydroxyl, carboxyl, amino, and carbonyl groups were identified as instrumental in Cr^6+^ binding. Oh et al. (2009) utilized FT-IR spectroscopy to validate interactions between functional groups on the cell wall of *Pseudomonas stutzeri* and heavy metals Pb^2+^, Cd^2+^, and Cu^2+^. The intricate mechanisms underlying metal removal by microorganisms were classified into three groups, involving biosorption of metal ions onto the cell surface, intracellular uptake of metal ions, and chemical transformation of metal ions by microorganisms. Various studies highlighted the role of functional groups, such as carboxylic, hydroxyl, and amino groups, in the biosorption process (Pardo et al., 2003; Oh et al., 2009; George et al., 2012; Michalak et al., 2013; Gu and Lan, 2021; Pagnucco et al., 2023).

Saranya et al. (2018) employed FT-IR spectroscopy to study the biosorption of multi-heavy metals by *Cronobacter muytjensii* KSCAS2. The analysis of both control and mixed heavy metals-treated bacterial cultures through FT-IR revealed that the biosorption capacity was influenced by the functional groups present on the active sites of bacterial cells, as confirmed by SEM results. Moreover, Dhanwal et al. (2018) examined FT-IR spectra of bacterial cells isolated from contaminated soil, identifying various absorption peaks indicating the involvement of multiple functional groups. These findings emphasize the valuable role of FT-IR spectroscopy in elucidating the intricate mechanisms of microbial biosorption processes and the significance of different functional groups. FT-IR spectroscopy aids in characterizing biosorption sites and the affinity of microorganisms for specific metals, providing insights into the nature of the binding sites. Furthermore, the quantification of metal-induced spectral changes can offer information on metal affinities and competitive binding behavior, enabling the optimization of biosorption processes and prediction of metal removal efficiency (Dhanwal et al., 2018; Pagnucco et al., 2023).

Pagnucco et al. (2023) note that biosorption can impact the biochemical composition of microbial cells. FT-IR spectroscopy, as highlighted by Ojeda and Dittrich (2012), enables the monitoring of changes in biomolecule vibrational modes, revealing alterations in cellular structure and function caused by metal stress. Analyzing FT-IR spectra provides valuable insights into the effects of biosorption on microbial physiology and viability (Meng et al., 2023). This technique finds applications in diverse metal-contaminated environments, including industrial effluents and mine tailings, as discussed by Tiquia-Arashiro (2012), Mudziwapasi et al. (2018), and Sundararaju et al. (2022). FT-IR-based analyses aid in optimizing biosorption conditions, understanding the influence of environmental parameters on metal uptake, and developing specialized microbial biosorbents for specific metals. The integration of FT-IR with other analytical techniques, such as X-ray absorption spectroscopy and microscopy, promises a holistic understanding of metal-microbe interactions across different scales.

The use of FT-IR spectroscopy in microbial biosorption studies of metals is valuable but not without limitations. While it enables the investigation of molecular-level changes during metal binding on microbial cell surfaces, the limited spatial resolution of FT-IR restricts the detailed examination of microscopic variations within cell wall structures, potentially overlooking crucial details. Moreover, while FT-IR provides valuable insights into the chemical composition of cell walls, it may not offer the high-resolution structural details required for understanding complex interactions between cell wall components and external factors. The integration of FT-IR alongside complementary analytical methods such as scanning electron microscopy with energy dispersive X-ray spectroscopy (SEM/EDX) and Transmission Electron Microscopy with EDX shows promise in comprehensively unraveling the complexities of metal-microbe interactions at different scales (Pagnucco et al., 2023).

### 5.5. Microbial degradation of organic pollutants

The application of FT-IR spectroscopy in the study of microbial interactions with organic pollutants, such as polycyclic aromatic hydrocarbons (PAHs), pesticides, and emerging contaminants like pharmaceuticals and personal care products, has garnered significant attention (Obinaju and Martin, 2016; Chakraborty and Das, 2017; Monga et al., 2021; Kumar et al., 2022; Cáceda Quiroz et al., 2023; Chaudhary et al., 2023; Hosseini et al., 2023; Nor et al., 2023; Table 10).

**Table 10:** Applications of FT-IR in microbial degradation of organic pollutants.

Research goal	Microorganism	Data analysis	Reference
Use a novel approach of sequestering PAH, phenanthrene, onto a solid carbon matrix bioanode in a microbial fuel cell (MFC) to assess its biodegradation coupled with power generation.	Microbial communities	**-**	Sharma et al. (2020)
Determine effective biotreatment processes of aqueous methylparathion by competent potential indigenous strains of bacterium (*Pseudomonas aeruginosa* mpd5) and fungus (*Fusarium* spp. mpd1).	*Pseudomonas aeruginosa*, *Fusarium* spp.	**-**	Krishnaswamy (2021)
Investigate the degradation of amphotericin B (AmB) with simultaneous wastewater treatment in a microbial peroxide producing cell (MPPC)	Microbial communities	-	Chang and Gupta (2022)
Evaluate the biosurfactant property of an extremophilic microbe *Bacillus cereus* KH1 in decolorizing the textile dyes.	*Bacillus cereus* KH1	-	Nor et al. (2023)
Use FTIR spectroscopy to study the functional groups of compounds made during the biodegradation of alkaline cyanide to figure out the specific biochemical mechanisms and pathways involved	*Bacillus subtilis* strain TT10s	-	Cáceda Quiroz et al. (2023)
Investigate the capability and efficiency of co-contaminant bioremediation of phenol and tellurite as two models of aromatic compounds and metalloid oxyanions.	*Lysinibacillus* sp. EBL303	Kolmogorov–Smirnov and Levene’s test, Analysis of variance (ANOVA)	Hosseini et al. (2023)
Evaluate volatile organic carbon (VOCs) removal efficiencies of VOC-degrading bacteria using diffusion bioreactor	*Pseudomonas* sp. DKR-23 and *Rhodococcus* sp. Korf-18	-	Chaudhary et al. (2023)

Sharma et al. (2020) introduced a pioneering bioelectroremediation strategy for efficiently eliminating phenanthrene, a significant polycyclic aromatic hydrocarbon (PAH) pollutant, while concurrently generating power. This technique not only captured electrons released during microbial metabolism but also fostered the growth of microbial communities specializing in PAH degradation, including *Pseudomonas*, *Thauera*, and *Rhodococcus*. The researchers employed FT-IR to track the biodegradation process, identifying the presence of phenanthrene through distinct C–H out-of-plane stretching vibrations at approximately 730 cm^−1^ in the phenanthrene-loaded electrodes. Subsequent analysis after 50 days revealed the disappearance of the ∼730 cm^−1^ band, suggesting the successful breakdown or elimination of phenanthrene from the sample. Moreover, a new major band emerged in the 1,170–980 cm^−1^ range, with a peak at approximately 1,030 cm^−1^, indicating the potential involvement of various functional groups within the polysaccharides due to the biofilms formed by the microorganisms. These FT-IR findings provided robust evidence of phenanthrene degradation by the microbial communities.

Krishnaswamy (2021) delved into the efficient biotreatment techniques for methyl parathion, an agricultural pesticide designed to combat crop-damaging insects, using native strains of *Pseudomonas aeruginosa* and *Fusarium* spp. Through specific biotransformation experiments, various biodegradation compounds were identified. Gas chromatography–mass spectrometry (GCMS) analysis of the transformed compounds by the bacteria unveiled the presence of p-nitrophenol, dimethylaminophenol, and other derivatives. The study extensively utilized FT-IR spectroscopy to examine the structural changes of methyl parathion and its products. The spectra highlighted distinct bands corresponding to specific functional groups, including 2,955 cm^−1^ for CH_3_, 837 cm^−1^ for CH in the outer plane of the aromatic ring, and 1,641 cm^−1^ for C=C. Furthermore, the presence of bands around 1,346 cm^−1^ and 1,047 cm^−1^ was associated with the aromatic NO_2_ group and the P–OCH_3_ group, respectively. Additionally, the phosphate group was identified through bands at 576 cm^−1^ and 1,036 cm^−1^ for P–O stretching and bending vibrations, and at 1,036 cm^−1^ and 767 cm^−1^ for P–O–C and P = S stretching, respectively. The spectral analysis provided crucial insights into the structural modifications occurring during the biotreatment processes.

Chang and Gupta (2022) demonstrated the degradation of Amphotericin B (AmB, an antifungal drug) in tandem with wastewater treatment within a Microbial Peroxide Producing Cell (MPPC). Two sets of MPPCs were employed for the oxidative degradation of AmB, one utilizing H_2_O_2_ and the other employing the microbial electro-Fenton process. FT-IR analysis of the treated samples unveiled the disappearance of characteristic bands such as the NH band at 1,556 cm^−1^ and the CH band in the Polyene ring at 3,358 cm^−1^. This indicated the disruption of multiple double bonds in the polyene structure, resulting in the breakdown of the lactone ring. Liquid chromatography quadrupole time-of-flight confirmed the structural changes by revealing shifts in retention time and peak area compared to native AmB, leading to the loss of its antifungal action. The absence of zones of inhibition in an antimicrobial susceptibility test against *Candida albicans* corroborated this finding.

FT-IR analysis of microbial degradation of organic pollutants faces several limitations due to the intricate nature of microbial samples. Distinguishing specific functional groups within diverse microbial communities can be difficult (Sharma et al., 2020; Chang and Gupta, 2022), leading to potential misinterpretation of data. Inconsistent sample preparation techniques, such as variations in drying procedures, can introduce errors and hinder data reliability. Instrumental limitations, including resolution and sensitivity, may impede the detection of subtle spectral changes in complex samples, affecting the accuracy of results. Interference from other organic and inorganic components in the sample matrix can obscure the identification of target pollutants and their interactions with the microbial biomass. To address the challenges associated with FT-IR analysis of microbial degradation of organic pollutants, several potential solutions can be implemented. Advanced data processing methods such as peak fitting, baseline correction, and deconvolution can assist in discerning specific functional groups in complex microbial samples. Ensuring standardized sample preparation protocols, including consistent drying, presentation, and handling techniques, can reduce variability and ensure consistent spectral data. Regular calibration of FT-IR instruments, along with rigorous quality control measures, can improve the precision and accuracy of the analysis. Establishing comprehensive spectral databases covering diverse microbial samples and organic pollutants can aid in the precise identification of spectral signatures and facilitate data interpretation. Performing thorough sample matrix analyses and utilizing advanced spectral analysis techniques to isolate target pollutants from background noise and overlapping signals can help minimize spectral interference. Collaboration among experts in microbiology, chemistry, and spectroscopy can drive the development of integrated approaches, promoting innovative solutions for dependable FT-IR analysis and deeper insights into the intricate dynamics of microbial interactions with organic pollutants.

## 6. Challenges

The applications of Fourier Transform-Infrared (FT-IR) spectroscopy have ushered in new possibilities for understanding microbial composition, functions, and interactions and have brought about significant advancements in our understanding of microbial communities and their interactions within complex ecosystems. However, these applications are not without their challenges. Several key challenges need to be addressed to harness the full potential of FT-IR in advancing microbiological research.

1. *Sample complexity and heterogeneity:* Microbial communities are composed of diverse microorganisms with distinct cellular compositions. FT-IR spectra can be influenced by variations in biomass, cell size, and cellular content. Discriminating between different functional groups and their contributions within mixed microbial communities remains a challenge. In complex mixtures, functional groups can produce overlapping peaks, making it difficult to deconvolute and accurately identify individual components. This requires careful spectral interpretation and, in some cases, additional techniques to separate overlapping signals. Advances in single-cell FT-IR spectroscopy (Doherty et al., 2019) may facilitate the precise analysis of the biochemical constituents and functional attributes of individual microbial cells, enabling researchers to gain a comprehensive understanding of the intricate responses and behaviors exhibited by diverse microorganisms within complex microbial communities.
2. *Quantitative analysis:* While FT-IR provides qualitative insights into microbial composition and functional groups, quantitative analysis remains a challenge. Factors like sample thickness, path length, and scattering can affect the accuracy of quantitative measurements (Wenning and Scherer, 2013; Watanabe et al., 2023). The development of robust calibration techniques and the establishment of reference standards are pivotal in ensuring the precise quantification of specific biomolecules and metabolites within microbial samples. These calibration methods are essential for translating the spectral data obtained from FT-IR analysis into quantifiable and meaningful information, enabling researchers to accurately assess the concentration of microbial biomass, monitor the production of key metabolic products, and quantify the levels of pollutants within diverse microbial systems. Overcoming the challenges associated with quantitative analysis in FT-IR spectroscopy is vital for enhancing the applicability and reliability of this analytical tool in the field of microbiology research.
3. *Data interpretation and standardization:* Interpreting FT-IR spectra is a multidisciplinary task that demands expertise in both microbiology and spectroscopy. The complexity of microbial samples, which may contain a multitude of functional groups and biomolecules, can make deciphering the spectral information daunting. Standardization of protocols for sample preparation, data acquisition, and analysis is crucial to ensure consistent and comparable results across studies. Establishing clear guidelines for data interpretation will enhance the reliability and reproducibility of FT-IR-based microbiological studies.
4. *Multivariate analysis and data handling:* FT-IR spectra generate large datasets containing intricate spectral information. Analyzing these datasets requires advanced multivariate analysis techniques, such as PCA and clustering algorithms like PCA, CVA, and SIMCA. Developing robust algorithms capable of extracting meaningful information from complex spectra is essential to unveil patterns, trends, and relationships within microbial data. Combining FT-IR with machine learning approaches could enhance our ability to distinguish microbial species, strains, and functional states accurately.

## 7. Future prospects

The challenges posed by FT-IR in microbiology also present opportunities for innovation and advancement. The future prospects of FT-IR applications in microbiology are promising and hold the potential to reshape how we perceive and study microorganisms.

1. *Development of comprehensive and standardized FT-IR libraries for the identification of bacteria and fungi:* While the utilization of FT-IR spectroscopy for microbial identification has shown considerable potential, the establishment of extensive libraries could significantly enhance the accuracy, efficiency, and reliability of this identification method. One of the primary objectives in future research could be the creation of a universal, exhaustive, and well-curated spectral database that encompasses a diverse range of bacterial and fungal species. This library should include spectra obtained under varying growth conditions, substrates, and environmental factors to ensure robustness and applicability across different settings and contexts. To achieve this, collaborative efforts among researchers, microbiologists, and spectroscopy experts are essential. It is crucial to encourage data sharing and collaboration between different research groups and institutions to compile a diverse and representative collection of spectra. Furthermore, the inclusion of strains with clinical relevance, such as those associated with antibiotic resistance or pathogenicity, would enhance the practical utility of the library in clinical and diagnostic settings. In the process of building these libraries, it is important to consider the standardization of experimental protocols and data acquisition parameters to ensure consistency and comparability across different studies and laboratories. This includes defining standardized procedures for sample preparation, spectral acquisition, and data analysis to minimize variations and improve the reproducibility of results. Integration of advanced data analysis techniques, such as machine learning algorithms and pattern recognition methods, would further enhance the capabilities of FT-IR libraries for microbial identification. Leveraging these tools can enable the development of automated and efficient identification systems capable of handling large datasets and complex spectral information, thereby facilitating rapid and accurate microbial identification. Incorporating metadata, such as strain information, growth conditions, and environmental factors, into the spectral database would provide valuable insights into the effects of various parameters on microbial spectra, enabling a more comprehensive understanding of the relationships between microbial physiology and spectral fingerprints.
2. *Integration of advanced analytical techniques:* The integration of FT-IR with other advanced analytical techniques, such as Raman spectroscopy, mass spectrometry, and genomics, represents a transformative approach in the study of microbial systems, enabling a comprehensive and multi-dimensional analysis of their intricate functionalities. By merging the capabilities of FT-IR with Raman spectroscopy, researchers can attain a more detailed understanding of molecular structures and spatial distributions within heterogeneous microbial samples. Simultaneously, the fusion of FT-IR with mass spectrometry allows for the identification and quantification of specific biomolecules and metabolites, shedding light on complex metabolic pathways and molecular interactions. Additionally, coupling FT-IR with genomics and molecular biology techniques provides insights into the genetic underpinnings of microbial characteristics, facilitating the correlation of spectral data with specific genetic signatures and facilitating the identification of key biomarkers associated with distinct microbial phenotypes and environmental adaptations. This integrative analytical approach not only enhances microbial identification accuracy but also fosters a deeper comprehension of microbial behavior, offering significant implications for diverse fields such as biotechnology, medicine, environmental science, and bioprocess engineering.
3. *High-throughput and single-cell analysis*: Technological advancements are paving the way for high-throughput FT-IR analysis, enabling rapid screening of microbial communities and dynamic responses. Additionally, single-cell FT-IR spectroscopy has the potential to achieve single-cell resolution. Recent advancements in the technology have enabled the development of methods for analyzing individual microbial cells (Saulou et al., 2013; Muhamadali et al., 2015; Harrison and Berry, 2017; Meneghel et al., 2020). The single-cell FT-IR approach can facilitate the study of diverse microbial properties, including metabolic activities, cellular responses to environmental stimuli, and the identification of specific biomolecules within a single microbial cell. This level of resolution is valuable for understanding the intricacies of microbial physiology and behavior at a microscale level, offering insights into cellular processes and enabling a more comprehensive understanding of microbial functionality.
4. *Real-time environmental monitoring:* Portable FT-IR systems could revolutionize environmental monitoring by enabling real-time, *in situ* analysis of microbial communities. Such technology would allow researchers to track microbial responses to changing environmental conditions, detect pollution events, and assess ecosystem health promptly. Mitigating the interference of exogenous and endogenous substances remains a significant concern in environmental samples. Addressing this challenge necessitates the implementation of advanced data processing algorithms, signal deconvolution methods, and spectral library expansion to enable the accurate discrimination of target compounds from interfering substances. For example, comprehensive reference libraries containing spectral signatures of known exogenous and endogenous interfering substances can be established. This approach allows for the comparison of sample spectra with the reference database, aiding in the identification and correction of potential interferences during data analysis. Multivariate analysis techniques such as PCA and partial least squares regression (PLSR) to discern and account for the influence of interfering substances on the FT-IR spectra. These statistical methods enable the extraction of relevant spectral information and the identification of specific spectral patterns associated with target analytes, thereby reducing the impact of interferences. Moreover, the integration of complementary analytical techniques, such as chromatography and mass spectrometry, can enhance the selectivity and sensitivity of FT-IR in real-time environmental monitoring, allowing for the effective identification and quantification of specific pollutants amidst complex environmental matrices. These strategies, coupled with rigorous calibration and validation processes, can significantly contribute to minimizing the impact of interference and ensuring the reliability and accuracy of real-time environmental monitoring using FT-IR spectroscopy.
5. *Functional metagenomics integration:* Integrating FT-IR data with functional metagenomics can link microbial metabolic activities to spectral changes, providing a deeper understanding of microbial contributions to ecosystem functions. This integration could elucidate the functional roles of specific microbial taxa in biogeochemical cycles. It can also facilitate the establishment of direct correlations between the spectral signatures captured by FT-IR and the underlying genetic mechanisms governing microbial functions.
6. *Biosorption and bioremediation strategies:* Expanding FT-IR-based studies on microbial biosorption holds promise for developing efficient bioremediation strategies for metal removal. By utilizing FT-IR spectroscopy, researchers can investigate the molecular interactions between microorganisms and various contaminants, enabling a comprehensive understanding of biosorption mechanisms and bioremediation processes. Understanding the molecular-level interactions between microorganisms and metals could lead to the design of tailored biosorbents with enhanced metal-binding capacities.
7. *Ecological modeling and predictive insights:* Integrating FT-IR data into ecological models can enhance our ability to predict microbial responses to environmental changes. For example, FT-IR spectroscopy assists in identifying key biomolecular signatures associated with microbial activities, enabling the development of predictive models that forecast ecosystem responses to environmental changes, such as shifts in nutrient availability, climate fluctuations, and anthropogenic disturbances. By coupling spectral information with ecological modeling approaches, researchers can gain insights into how microbial communities shape ecosystem dynamics and stability.

## 8. Conclusion

In conclusion, the challenges encountered in harnessing FT-IR in microbiology serve as catalysts for innovation and exploration of novel solutions. Addressing these challenges through interdisciplinary collaboration and technological advancements will pave the way for an exciting future in microbiological research. As FT-IR technology evolves and its integration with other techniques becomes more seamless, the scope of microbial analysis will expand, enabling us to uncover hidden insights into microbial communities, functions, and their roles in shaping our understanding of ecosystem dynamics and sustainability.

## Author contributions

AK: Formal analysis, Validation, Writing – original draft, Writing – review & editing. LA: Methodology, Validation, Writing – original draft, Writing – review & editing. OC: Methodology, Validation, Writing – original draft, Writing – review & editing. SO: Methodology, Validation, Writing – original draft, Writing – review & editing. HN: Validation, Writing – original draft, Writing – review & editing. DK: Writing – original draft, Writing – review & editing. KP: Validation, Resources, Writing – review & editing. XL: Methodology, Supervision, Validation, Writing – original draft, Writing – review & editing. ST-A: Conceptualization, Funding acquisition, Project administration, Resources, Supervision, Validation, Visualization, Writing – original draft, Writing – review & editing.

## References

[ref1] AlMasoud N. Muhamadali H. Chisanga M. AlRabiah H. Lima C. A. Goodacre R. (2021). Discrimination of bacteria using whole organism fingerprinting: the utility of modern physicochemical techniques for bacterial typing. Analyst 146, 770–788. doi: 10.1039/D0AN01482F, PMID: 33295358

[ref2] AlQadiri M. Al-Alami N. I. Lin M. Al-Holy M. Cavinato A. G. Rasco B. A. (2008). Studying of the bacterial growth phases using fourier transform infrared spectroscopy and multivariate analysis. J. Rapid Met. Autom. Microbiol. 16, 73–89. doi: 10.1111/j.1745-4581.2008.00117.x

[ref3] Alvarez-Ordóñez A. Mouwen D. J. López M. Prieto M. (2011). Fourier transform infrared spectroscopy as a tool to characterize molecular composition and stress response in foodborne pathogenic bacteria. J. Microbiol. Methods 84, 369–378. doi: 10.1016/j.mimet.2011.01.009, PMID: 21256893

[ref4] Andrei A. Ş. Robeson M. Baricz A. Coman C. Muntean V. Ionescu A.. (2015). Contrasting taxonomic stratification of microbial communities in two hypersaline meromictic lakes. ISME J. 9, 2642–2656. doi: 10.1038/ismej.2015.60, PMID: 25932617 PMC4817630

[ref5] Artz R. Chapman S. J. Campbell C. D. (2006). Substrate utilisation profiles of microbial communities in peat are depth dependent and correlate with whole soil FT-IR profiles. Soil Biol. Biochem. 38, 2958–2962. doi: 10.1016/j.soilbio.2006.04.017

[ref6] Ashajyothi M. Balamurugan A. Patel A. Krishnappa C. Kumar R. Kumar A. (2023). Cell wall polysaccharides of endophytic *Pseudomonas putida* elicit defense against rice blast disease. J. Appl. Microbiol. 134:lxac042. doi: 10.1093/jambio/lxac042, PMID: 36626745

[ref7] Aziz S. Ali M. I. Farooq U. Jamal A. Liu F. J. He H.. (2020). Enhanced bioremediation of diesel range hydrocarbons in soil using biochar made from organic wastes. Environ. Monit. Assess. 192:569. doi: 10.1007/s10661-020-08540-7, PMID: 32770276

[ref8] Baker M. J. Trevisan J. Bassan P. Bhargava R. Butler H. J. Dorling K. M.. (2014). Using Fourier transform IR spectroscopy to analyze biological materials. Nate Protocols. 9, 1771–1791. doi: 10.1038/nprot.2014.110, PMID: 24992094 PMC4480339

[ref9] Beekes M. Lasch P. Naumann D. (2007). Analytical applications of Fourier transform-infrared (FT-IR) spectroscopy in microbiology and prion research. Vet. Microbiol. 123, 305–319. doi: 10.1016/j.vetmic.2007.04.010, PMID: 17540519

[ref10] Berninger T. González López Ó. Bejarano A. Preininger C. Sessitsch A. (2018). Maintenance and assessment of cell viability in formulation of non-sporulating bacterial inoculants. Microb. Biotechnol. 11, 277–301. doi: 10.1111/1751-7915.12880, PMID: 29205959 PMC5812248

[ref11] Bhargava R. (2012). Infrared spectroscopic imaging: the next generation. Appl. Spectrosc. 66, 1091–1120. doi: 10.1366/12-06801, PMID: 23031693 PMC3756188

[ref12] Bombalska A. Mularczyk-Oliwa M. Kwaśny M. Włodarski M. Kaliszewski M. Kopczyński K.. (2011). Classification of the biological material with use of FT-IR spectroscopy and statistical analysis. Spectrochim. Acta A Mol. Biomol. Spectrosc. 78, 1221–1226. doi: 10.1016/j.saa.2010.10.025, PMID: 21257340

[ref13] Bosch A. Serra D. Prieto C. Schmitt J. Naumann D. Yantorno O. (2006). Characterization of *Bordetella pertussis* growing as biofilm by chemical analysis and FT-IR spectroscopy. Appl. Micro. Biotechnol. 71, 736–747. doi: 10.1007/s00253-005-0202-8, PMID: 16292646

[ref14] Bottum S. R. Teitsworth T. S. Han Q. Orr A. D. Park J. S. Jia X.. (2023). *In situ* attenuated Total reflectance infrared Spectroelectrochemistry (ATR-IR-SEC) for the characterization of molecular redox processes on surface-proximal doped silicon ATR crystal working electrodes. J. Phys. Chem. C. 127, 6690–6701. doi: 10.1021/acs.jpcc.2c08991

[ref15] Bowman N. Patel D. Sanchez A. Xu W. Alsaffar A. Tiquia-Arashiro S. M. (2018). Lead-resistant bacteria from Saint Clair River sediments and Pb removal in aqueous solutions. Appl. Microbiol. Biotechnol. 102, 2391–2398. doi: 10.1007/s00253-018-8772-4, PMID: 29354853

[ref16] Braissant O. Astasov-Frauenhoffer M. Waltimo T. Bonkat G. (2020). A review of methods to determine viability, vitality, and metabolic rates in microbiology. Front. Microbiol. 11:547458. doi: 10.3389/fmicb.2020.547458, PMID: 33281753 PMC7705206

[ref17] Brandes A. Brandl H. (2011). Detection and differentiation of bacterial spores in a mineral matrix by Fourier transform infrared spectroscopy (FT-IR) and chemometrical data treatment. BMC Biophys. 4, 1–8. doi: 10.1186/2046-1682-4-14, PMID: 21756333 PMC3155104

[ref18] Brito N. M. R. D. Lourenço F. R. (2021). Rapid identification of microbial contaminants in pharmaceutical products using a PCA/LDA-based FTIR-ATR method. Brazilian J. Pharm. Sci. 57:8899. doi: 10.1590/s2175-97902020000318899

[ref19] Cabugao K. G. M. Gushgari-Doyle S. Chacon S. S. Wu X. Bhattacharyya A. Bouskill N.. (2022). Characterizing natural organic matter transformations by microbial communities in terrestrial subsurface ecosystems: a critical review of analytical techniques and challenges. Front. Microbiol. 13:864895. doi: 10.3389/fmicb.2022.864895, PMID: 35602028 PMC9114703

[ref20] Cáceda Quiroz C. J. Fora Quispe G. L. Carpio Mamani M. Maraza Choque G. J. Sacari Sacari E. J. (2023). Cyanide bioremediation by *Bacillus subtilis* under alkaline conditions. Water 15:3645. doi: 10.3390/w15203645

[ref21] Câmara A. A. Jr. Nguyen T. D. Saurel R. Sandt C. Peltier C. Dujourdy L.. (2020). Biophysical stress responses of the yeast Lachancea thermotolerans during dehydration using synchrotron-FTIR microspectroscopy. Front. Microbiol. 11:899. doi: 10.3389/fmicb.2020.00899, PMID: 32477306 PMC7235352

[ref22] Chakraborty J. Das S. (2017). Application of spectroscopic techniques for monitoring microbial diversity and bioremediation. Appl. Spectr. Rev. 52, 1–38. doi: 10.1080/05704928.2016.1199028

[ref23] Chang C. Gupta P. (2022). *In-situ* degradation of amphotericin B in a microbial electrochemical cell containing wastewater. Chemosphere 309:136726. doi: 10.1016/j.chemosphere.2022.136726, PMID: 36209861

[ref24] Chaudhary D. K. Park J. H. Kim P. G. Ok Y. S. Hong Y. (2023). Enrichment cultivation of VOC-degrading bacteria using diffusion bioreactor and development of bacterial-immobilized biochar for VOC bioremediation. Environ. Pollut. 320:121089. doi: 10.1016/j.envpol.2023.121089, PMID: 36669717

[ref25] Cheah Y. T. Chan D. J. C. (2022). A methodological review on the characterization of microalgal biofilm and its extracellular polymeric substances. J. Appl. Microbiol. 132, 3490–3514. doi: 10.1111/jam.15455, PMID: 35061929

[ref26] Cheeseman S. Shaw Z. L. Vongsvivut J. Crawford R. J. Dupont M. F. Boyce K. J.. (2021). Analysis of pathogenic bacterial and yeast biofilms using the combination of synchrotron ATR-FTIR microspectroscopy and chemometric approaches. Molecules 26:3890. doi: 10.3390/molecules26133890, PMID: 34202224 PMC8271424

[ref27] Cho K. Zholi A. Frabutt D. Flood M. Floyd D. Tiquia S. M. (2012). Linking bacterial diversity and geochemistry of uranium-contaminated groundwater. Environ. Technol. 33, 1629–1640. doi: 10.1080/09593330.2011.641036, PMID: 22988623

[ref28] Cooper S. Bacterial growth and division: Biochemistry and regulation of prokaryotic and eukaryotic division cycles. (1991). Elsevier Science: New York, NY. 501.

[ref29] Corte L. Antonielli L. Roscini L. Fatichenti F. Cardinali G. (2011). Influence of cell parameters in Fourier transform infrared spectroscopy analysis of whole yeast cells. Analyst 136, 2339–2349. doi: 10.1039/c0an00515k, PMID: 21494743

[ref30] Corte L. Tiecco M. Roscini L. Germani R. Cardinali G. (2014). FT-IR analysis of the metabolomic stress response induced by N-alkyltropinium bromide surfactants in the yeasts Saccharomyces cerevisiae and *Candida albicans*. Colloids Surf B Biointer. 116, 761–771. doi: 10.1016/j.colsurfb.2014.01.054, PMID: 24582147

[ref31] Davis R. Deering A. Burgula Y. Mauer L. J. Reuhs B. L. (2012). Differentiation of live, dead and treated cells of *Escherichia coli* O157:H7 using FT-IR spectroscopy. J. Appl. Microbiol. 112, 743–751. doi: 10.1111/j.1365-2672.2011.05215.x, PMID: 22151262

[ref32] Dhanwal P. Kumar A. Dudeja S. Badgujar H. Chauhan R. Kumar A.. (2018). Biosorption of heavy metals from aqueous solution by bacteria isolated from contaminated soil. Water Environ. Res. 90, 424–430. doi: 10.2175/106143017X15131012152979, PMID: 29678213

[ref33] Di Martino P. (2018). Extracellular polymeric substances, a key element in understanding biofilm phenotype. AIMS Microbiol. 4, 274–288. doi: 10.3934/microbiol.2018.2.274, PMID: 31294215 PMC6604936

[ref34] Dimopoulou M. Kefalloniti V. Tsakanikas P. Papanikolaou S. Nychas G.-J. E. (2021). Assessing the biofilm formation capacity of the wine spoilage yeast Brettanomyces bruxellensis through FTIR spectroscopy. Microorganisms 9:587. doi: 10.3390/microorganisms9030587, PMID: 33809238 PMC7999561

[ref9001] Doherty J. Raoof A. Hussain A. Wolna M. Cinque G. Brown M.. (2019). Live single cell analysis using synchrotron FTIR microspectroscopy: development of a simple dynamic flow system for prolonged sample viability. Analyst. 144, 997–1007. doi: 10.1039/C8AN01566J 30403210

[ref35] Dong J. Zhang Z. Yu Z. Dai X. Xu X. Alvarez P. J.. (2017). Evolution and functional analysis of extracellular polymeric substances during the granulation of aerobic sludge used to treat p-chloroaniline wastewater. Chem. Engin. J. 330, 596–604. doi: 10.1016/j.cej.2017.07.174

[ref36] Douterelo I. Boxall J. B. Deines P. Sekar R. Fish K. E. Biggs C. A. (2014). Methodological approaches for studying the microbial ecology of drinking water distribution systems. Water Res. 65, 134–156. doi: 10.1016/j.watres.2014.07.008, PMID: 25105587

[ref37] Duncan R. J. Petrou K. (2022). Biomolecular composition of sea ice microalgae and its influence on marine biogeochemical cycling and carbon transfer through polar marine food webs. Geosciences 12:38. doi: 10.3390/geosciences12010038

[ref38] Dziuba B. Babuchowski A. Nałęcz D. Niklewicz M. (2007). Identification of lactic acid bacteria using FT-IR spectroscopy and cluster analysis. Int. Dairy J. 17, 183–189. doi: 10.1016/j.idairyj.2006.02.013

[ref39] Edwards A. Mur L. A. Girdwood S. E. Anesio A. M. Stibal M. Rassner S. M.. (2014). Coupled cryoconite ecosystem structure-function relationships are revealed by comparing bacterial communities in alpine and Arctic glaciers. FEMS Microbiol. Ecol. 89, 222–237. doi: 10.1111/1574-6941.12283, PMID: 24433483

[ref40] El-Azazy M. El-Shafie A. S. Al-Saad K. (2021). Introductory chapter: infrared spectroscopy-principles and applications. Infrared spectroscopy-perspectives and applications. Intech Open. 1–13. doi: 10.5772/intechopen.109139

[ref41] El-Bialy H. A. El-Gamal M. S. Elsayed M. A. Saudi H. A. Khalifa M. A. (2019). Microbial melanin physiology under stress conditions and gamma radiation protection studies. Radiat. Phys. Chem. 162, 178–186. doi: 10.1016/j.radphyschem.2019.05.002

[ref42] Elzinga E. J. Huang J. H. Chorover J. Kretzschmar R. (2012). ATR-FTIR spectroscopy study of the influence of pH and contact time on the adhesion of *Shewanella putrefaciens* bacterial cells to the surface of hematite. Environ. Sci. Technol. 46, 12848–12855. doi: 10.1021/es303318y, PMID: 23136883

[ref43] Fadlelmoula A. Pinho D. Carvalho V. H. Catarino S. O. Minas G. (2022). Fourier transform infrared (FTIR) spectroscopy to analyse human blood over the last 20 years: a review towards lab-on-a-Chip devices. Micromachines 13:187. doi: 10.3390/mi13020187, PMID: 35208311 PMC8879834

[ref44] Feng B. Shi H. Xu F. Hu F. He J. Yang H.. (2020). FTIR-assisted MALDI-TOF MS for the identification and typing of bacteria. Anal. Chim. Acta 1111, 75–82. doi: 10.1016/j.aca.2020.03.037, PMID: 32312399

[ref45] Flood M. Frabutt D. Floyd D. Powers E. U. Devol A. Tiquia-Arashiro S. M. (2015). Ammonia-oxidizing bacteria and archaea in sediments of the Gulf of Mexico. Environ. Technol. 36, 124–135. doi: 10.1080/09593330.2014.942385, PMID: 25409591

[ref46] Franco-Duarte R. Černáková L. Kadam S. Kaushik K. Salehi B. Bevilacqua A.. (2019). Advances in chemical and biological methods to identify microorganisms—from past to present. Microorganisms 7:130. doi: 10.3390/microorganisms7050130, PMID: 31086084 PMC6560418

[ref47] Galichet A. Sockalingum G. D. Belarbi A. Manfait M. (2001). FT-IR spectroscopic analysis of *Saccharomyces cerevisiae* cell walls: study of an anomalous strain exhibiting a pink-colored cell phenotype. FEMS Microbiol. Lett. 197, 179–186. doi: 10.1111/j.1574-6968.2001.tb10601.x, PMID: 11313132

[ref48] Gentile M. Yan T. Tiquia S. M. Fields M. W. Nyman J. Zhou J.. (2006). Stability and resilience in a denitrifying fluidized bed reactor. Microb. Ecol. 52, 311–321. doi: 10.1007/s00248-006-9024-1, PMID: 16874554

[ref49] Geoghegan M. Andrews J. S. Biggs C. A. Eboigbodin K. E. Elliott D. R. Rolfe S.. (2008). The polymer physics and chemistry of microbial cell attachment and adhesion. Faraday Discuss. 139, 85–103. doi: 10.1039/B717046G, PMID: 19048992

[ref50] George B. Nirmal Kumar J. I. Kumar R. N. Sajish P. R. (2012). Biosorption potentiality of living aspergillus Niger Tiegh in removing heavy metal from aqueous solution. Biorem. J. 16, 195–203. doi: 10.1080/10889868.2012.731442

[ref51] Gieroba B. Krysa M. Wojtowicz K. Wiater A. Pleszczyńska M. Tomczyk M.. (2020). The FT-IR and Raman spectroscopies as tools for biofilm characterization created by cariogenic streptococci. Int. J. Mol. Sci. 21:3811. doi: 10.3390/ijms21113811, PMID: 32471277 PMC7313032

[ref52] Glassford S. E. Byrne B. Kazarian S. G. (2013). Recent applications of ATR FTIR spectroscopy and imaging to proteins. Biochim et Biophys. Acta-Proteins Proteomics 1834, 2849–2858. doi: 10.1016/j.bbapap.2013.07.015, PMID: 23928299

[ref53] Gong M. Yang G. Zhuang L. Zeng E. Y. (2019). Microbial biofilm formation and community structure on low-density polyethylene microparticles in lake water microcosms. Environ. Pollut. 252, 94–102. doi: 10.1016/j.envpol.2019.05.090, PMID: 31146243

[ref54] Goodacre R. Shann B. Gilbert R. J. Timmins E. M. McGovern A. C. Alsberg B. K.. (2000). Detection of the dipicolinic acid biomarker in Bacillus spores using curie-point pyrolysis mass spectrometry and Fourier transform infrared spectroscopy. Anal. Chem. 72, 119–127. doi: 10.1021/ac990661i 10655643

[ref55] Grace C. E. E. Lakshmi P. K. Meenakshi S. Vaidyanathan S. Srisudha S. Mary M. B. (2020). Biomolecular transitions and lipid accumulation in green microalgae monitored by FT-IR and Raman analysis. Spectrochimica Acta Part A 224:117382. doi: 10.1016/j.saa.2019.117382, PMID: 31357053

[ref56] Griffiths P. R. (1983). Fourier transform infrared spectrometry. Science 222, 297–302. doi: 10.1126/science.6623077 6623077

[ref57] Gu S. Lan C. Q. (2021). Biosorption of heavy metal ions by green alga *Neochloris oleoabundans*: effects of metal ion properties and cell wall structure. J. Haz. Mat. 418:126336. doi: 10.1016/j.jhazmat.2021.126336, PMID: 34329013

[ref58] Gutiérrez-Corona J. F. Romo-Rodríguez P. Santos-Escobar F. Espino-Saldaña A. E. Hernández-Escoto H. (2016). Microbial interactions with chromium: basic biological processes and applications in environmental biotechnology. World J. Microbiol. Biotechnol. 32, 191–199. doi: 10.1007/s11274-016-2150-0, PMID: 27718146

[ref59] Harrison J. P. Berry D. (2017). Vibrational spectroscopy for imaging single microbial cells in complex biological samples. Front. Microbiol. 8:675. doi: 10.3389/fmicb.2017.00675, PMID: 28450860 PMC5390015

[ref60] He Z. Liu Y. Kim H. J. Tewolde H. Zhang H. (2022). Fourier transform infrared spectral features of plant biomass components during cotton organ development and their biological implications. J. Cotton Res. 5:11. doi: 10.1186/s42397-022-00117-8

[ref61] Helm D. Labischinski H. Schallehn G. (1991). Classification and identification of bacteria by Fourier-transform IR spectroscopy. J. Gen. Microbiol. 137, 69–79. doi: 10.1099/00221287-137-1-69 1710644

[ref62] Hosseini F. Lashani E. Moghimi H. (2023). Simultaneous bioremediation of phenol and tellurite by Lysinibacillus sp. EBL303 and characterization of biosynthesized Te nanoparticles. Sci. Rep. 13:1243. doi: 10.1038/s41598-023-28468-5, PMID: 36690691 PMC9870877

[ref63] Huang G. Ng T. W. An T. Li G. Wang B. Wu D.. (2017). Interaction between bacterial cell membranes and nano-TiO_2_ revealed by two-dimensional FT-IR correlation spectroscopy using bacterial ghost as a model cell envelope. Water Res. 118, 104–113. doi: 10.1016/j.watres.2017.04.023 28414961

[ref64] Huang T. Zhang W. Zhang F. Feng W. Li C. Zhang Y. (2020). The role of microbial community composition in controlling soil respiration after organic matter addition revealed by FT-IR spectroscopy. Soil Biol. Biochem. 11:107752. doi: 10.1371/journal.pone.0165448, PMID: 27798702 PMC5087920

[ref65] Igisu M. Takai K. Ueno Y. Nishizawa M. Nunoura T. Hirai M.. (2012). Domain-level identification and quantification of relative prokaryotic cell abundance in microbial communities by Micro-FTIR spectroscopy. Environ. Microbiol. Rep. 4, 42–49. doi: 10.1111/j.1758-2229.2011.00277.x, PMID: 23757228

[ref66] Jansson M. M. Kögler M. Hörkkö S. Ala-Kokko T. Rieppo L. (2023). Vibrational spectroscopy and its future applications in microbiology. Appl. Spect. Rev. 58, 132–158. doi: 10.1080/05704928.2021.1942894

[ref67] Jiang W. Saxena A. Song B. Ward B. B. Beveridge T. J. Myneni S. C. B. (2004). Elucidation of functional groups on gram-positive and gram-negative bacterial surfaces using infrared spectroscopy. Langmuir 20, 11433–11442. doi: 10.1021/la049043, PMID: 15595767

[ref68] Kamnev A. A. (2008). FT-IR spectroscopic studies of bacterial cellular responses to environmental factors, plant-bacterial interactions and signalling. Spectroscopy 22, 83–95. doi: 10.3233/SPE-2008-0329

[ref69] Kamnev A. A. Dyatlova Y. A. Kenzhegulov O. A. Fedonenko Y. P. Evstigneeva S. S. Tugarova A. V. (2023). Fourier transform infrared (FT-IR) spectroscopic study of biofilms formed by the Rhizobacterium Azospirillum baldaniorum Sp245: aspects of methodology and matrix composition. Molecules 28:1949. doi: 10.3390/molecules28041949, PMID: 36838937 PMC9962177

[ref70] Kamnev A. A. Tugarova A. V. Antonyuk L. P. Tarantilis P. A. Polissiou M. G. Gardiner P. H. (2005). Effects of heavy metals on plant-associated rhizobacteria: comparison of endophytic and non-endophytic strains of *Azospirillum brasilense*. J. Trace Elem. Med. Biol. 19, 91–95. doi: 10.1016/j.jtemb.2005.03.002, PMID: 16240678

[ref71] Kochan K. Lai E. Richardson Z. Nethercott C. Peleg A. Y. Heraud P.. (2020). Vibrational spectroscopy as a sensitive probe for the chemistry of intra-phase bacterial growth. Sensors 20:3452. doi: 10.3390/s20123452, PMID: 32570941 PMC7348983

[ref72] Koczoń P. Hołaj-Krzak J. T. Palani B. K. Bolewski T. Dąbrowski J. Bartyzel B. J.. (2023). The analytical possibilities of FT-IR spectroscopy powered by vibrating molecules. Int. J. Mol. Sci. 24:1013. doi: 10.3390/ijms24021013, PMID: 36674526 PMC9860999

[ref73] Krishnaswamy U. (2021). GCMS and FT-IR spectral analysis of aqueous methylparathion biotransformation by the microbial mpd strains of Pseudomonas aeruginosa and fusarium spp. Arch. Microbiol. 203, 5763–5782. doi: 10.1007/s00203-021-02520-2, PMID: 34510232

[ref74] Kumar S. S. Ghosh A. R. (2019). Assessment of bacterial viability: a comprehensive review on recent advances and challenges. Microbiology 165, 593–610. doi: 10.1099/mic.0.000786, PMID: 30843781

[ref75] Kumar R. Vuppaladadiyam A. K. Antunes E. Whelan A. Fearon R. Sheehan M.. (2022). Emerging contaminants in biosolids: presence, fate and analytical techniques. Emerg. Cont. 8, 162–194. doi: 10.1016/j.emcon.2022.03.004

[ref76] Lasch P. Beyer W. Nattermann H. Stämmler M. Naumann D. (2004). Discrimination of *Campylobacter jejuni* strains by using polychromatic Fourier-transform infrared spectroscopy and multivariate statistics. Appl. Environ. Microbiol. 71, 4318–4324. doi: 10.1128/AEM.71.8.4318-4324.2005, PMID: 16085819 PMC1183312

[ref77] Lilo T. Morais C. Shenton C. Ray A. Gurusinghe N. (2022). Revising Fourier-transform infrared (FT-IR) and Raman spectroscopy towards brain cancer detection. Photodiag Photodyn Ther. 38:102785. doi: 10.1016/j.pdpdt.2022.102785, PMID: 35231616

[ref78] Lin S. F. Schraft H. Griffiths M. W. (1998). Identification of *Bacillus cereus* by Fourier transform infrared spectroscopy (FT-IR). J. Food Prot. 61, 921–923. doi: 10.4315/0362-028x-61.7.921, PMID: 9678183

[ref79] Lin X. Tfaily M. M. Steinweg J. M. Chanton P. Esson K. Yang Z. K.. (2014). Microbial community stratification linked to utilization of carbohydrates and phosphorus limitation in a boreal peatland at Marcell experimental Forest, Minnesota, USA. Appl. Environ. Microbiol. 80, 3518–3530. doi: 10.1128/AEM.00205-14, PMID: 24682300 PMC4018854

[ref80] Liu X. Tiquia S. M. Holguin G. Wu L. Nold S. C. Devol A. H.. (2003). Molecular diversity of denitrifying genes in continental margin sediments within the oxygen-deficient zone off the Pacific coast of Mexico. Appl. Environ. Microbiol. 69, 3549–3560. doi: 10.1128/AEM.69.6.3549-3560.2003, PMID: 12788762 PMC161474

[ref82] Maquelin K. Choo-Smith L. P. Endtz H. P. Bruining H. A. Puppels G. J. Naumann D. (2000). Rapid identification of Candida species by confocal Raman microspectroscopy. J. Clin. Microbiol. 40, 594–600. doi: 10.1128/JCM.40.2.594-600.2002, PMID: 11825976 PMC153356

[ref83] Marques J. Ares A. Costa J. Marques M. P. M. de Carvalho L. A. E. B. Bessa F. (2023). Plastisphere assemblages differ from the surrounding bacterial communities in transitional coastal environments. Sci. Total Environ. 869:161703. doi: 10.1016/j.scitotenv.2023.161703, PMID: 36708826

[ref84] Martak D. Valot B. Sauget M. Cholley P. Thouverez M. Bertrand X.. (2019). Fourier-transform infrared spectroscopy can quickly type gram-negative Bacilli responsible for hospital outbreaks. Front. Microbiol. 10:1440. doi: 10.3389/fmicb.2019.01440, PMID: 31293559 PMC6606786

[ref85] McKindles K. M. Tiquia-Arashiro S. M. (2012). “Functional gene arrays for analysis of microbial communities on ocean platform” in Molecular biological Technologies for Ocean Sensing. Springer Protocols Handbooks. ed. Tiquia-Arashiro S. (Totowa, NJ: Humana Press)

[ref86] Melin A. M. Perromat A. Déléris G. (2001). Sensitivity of *Deinococcus radiodurans* to γ-irradiation: a novel approach by Fourier transform infrared spectroscopy. Arch. Biochem. Biophys. 394, 265–274. doi: 10.1006/abbi.2001.2533, PMID: 11594741

[ref87] Meneghel J. Passot S. Jamme F. Lefrançois S. Lieben P. Dumas P.. (2020). FT-IR micro-spectroscopy using synchrotron-based and thermal source-based radiation for probing live bacteria. Anal. Bioanal. Chem. 412, 7049–7061. doi: 10.1007/s00216-020-02835-x, PMID: 32839857

[ref88] Meng G. Liu J. Ma J. Liu X. Zhang F. Guo Y.. (2023). Biosorption and bioreduction of aqueous chromium (VI) by different Spirulina strains. FEMS Microbiol. Lett. 370:fnad070. doi: 10.1093/femsle/fnad070, PMID: 37475675

[ref89] Meyvisch P. Gurdebeke P. R. Vrielinck H. Neil Mertens K. Versteegh G. Louwye S. (2022). Attenuated Total reflection (ATR) Micro-Fourier transform infrared (Micro-FT-IR) spectroscopy to enhance repeatability and reproducibility of spectra derived from single specimen organic-walled dinoflagellate cysts. Appl. Spectrosc. 76, 235–254. doi: 10.1177/00037028211041172, PMID: 34494488

[ref90] Miao L. Wang P. Hou J. Yao Y. Liu Z. Liu S. (2019). Low concentrations of copper oxide nanoparticles alter microbial community structure and function of sediment biofilms. Sci. Total Environ. 653, 705–713. doi: 10.1016/j.scitotenv.2018.10.354, PMID: 30759596

[ref91] Michalak I. Chojnacka K. Witek-Krowiak A. (2013). State of the art for the biosorption process--a review. Appl. Biochem. Biotechnol. 170, 1389–1416. doi: 10.1007/s12010-013-0269-0 23666641 PMC3696181

[ref92] Moen B. Janbu A. O. Langsrud S. Langsrud O. Hobman J. L. Constantinidou C.. (2009). Global responses of *Escherichia coli* to adverse conditions determined by microarrays and FT-IR spectroscopy. Can. J. Microbiol. 55, 714–728. doi: 10.1139/w09-016, PMID: 19767843

[ref93] Moen B. Oust A. Langsrud Ø. Dorrell N. Marsden G. L. Hinds J.. (2005). Explorative multifactor approach for investigating global survival mechanisms of *Campylobacter jejuni* under environmental conditions. Appl. Environ. Microbiol. 71, 2086–2094. doi: 10.1128/AEM.71.4.2086-2094.2005, PMID: 15812042 PMC1082531

[ref94] Monga D. Kaur P. Singh B. (2021). Microbe mediated remediation of dyes, explosive waste and polyaromatic hydrocarbons, pesticides and pharmaceuticals. Curr. Res. Microb. Sci. 3:100092. doi: 10.1016/j.crmicr.2021.100092, PMID: 35005657 PMC8717453

[ref95] Mudziwapasi J. Peter M. Madzivire G. (2018). Biosorption of heavy metals in polluted water using microorganisms: progresses and challenges. J. Chem. 307, 135957–135911. doi: 10.1016/j.chemosphere.2022.135957

[ref96] Muhamadali H. Chisanga M. Subaihi A. Goodacre R. (2015). Combining Raman and FT-IR spectroscopy with quantitative isotopic labeling for differentiation of *E. coli* cells at community and single cell levels. Anal. Chem. 87, 4578–4586. doi: 10.1021/acs.analchem.5b00892, PMID: 25831066

[ref97] Mukherjee J. Ow S. Y. Noirel J. Biggs C. A. (2011). Quantitative protein expression and cell surface characteristics of *Escherichia coli* MG1655 biofilms. Proteomics 11, 339–351. doi: 10.1002/pmic.201000386, PMID: 21268264

[ref98] Nadtochenko V. A. Rincon A. G. Stanca S. E. Kiwi J. (2005). Dynamics of *E. coli* membrane cell peroxidation during TiO_2_ photocatalysis studied by ATR-FTIR spectroscopy and AFM microscopy. J. Photochem. Photobiol. A Chem. 169, 131–137. doi: 10.1016/j.jphotochem.2004.06.011

[ref99] Nandiyanto A. B. D. Oktiani R. Ragadhita R. (2019). How to read and interpret FT-IR spectroscopy of organic material. Indo. J. Sci. Technol. 4, 97–118. doi: 10.17509/ijost.v4i1.15806

[ref100] Naumann D. Helm D. Labischinski H. (2001). Microbiological characterizations by FT-IR spectroscopy. Nat. Protoc. 351, 81–82. doi: 10.1038/351081a0 1902911

[ref101] Naylor D. McClure R. Jansson J. (2022). Trends in microbial community composition and function by soil depth. Microorganisms 10:540. doi: 10.3390/microorganisms10030540, PMID: 35336115 PMC8954175

[ref102] Nguyen S. Ala F. Cardwell C. Cai D. McKindles K. M. Lotvola A.. (2013). Isolation and screening of carboxydotrophs isolated from composts and their potential for butanol synthesis. Environ. Technol. 34, 1995–2007. doi: 10.1080/09593330.2013.795987, PMID: 24350453

[ref103] Nor F. H. M. Abdullah S. Ibrahim Z. Nor M. H. M. Osman M. I. al Farraj D. A.. (2023). Role of extremophilic *Bacillus cereus* KH1 and its lipopeptide in treatment of organic pollutant in wastewater. Bioprocess Biosyst. Eng. 46, 381–391. doi: 10.1007/s00449-022-02749-1, PMID: 35779113

[ref105] Novais Â. Freitas A. R. Rodrigues C. Peixe L. (2019). Fourier transform infrared spectroscopy: unlocking fundamentals and prospects for bacterial strain typing. Eur. J. Clin. Microbiol. Infect. Dis. 38, 427–448. doi: 10.1007/s10096-018-3431-3, PMID: 30483997

[ref106] Oberbeckmann S. Loeder M. Gerdts G. Osborn A. M. (2014). Spatial and seasonal variation in diversity and structure of microbial biofilms on marine plastics in northern European waters. FEMS Microbiol. Ecol. 90, 478–492. doi: 10.1111/1574-6941.12409, PMID: 25109340

[ref107] Obinaju B. E. Martin F. L. (2016). ATR-FTIR spectroscopy reveals polycyclic aromatic hydrocarbon contamination despite relatively pristine site characteristics: results of a field study in the Niger Delta. Environ. Int. 89-90, 93–101. doi: 10.1016/j.envint.2016.01.012, PMID: 26826366

[ref108] Oest A. Fenner M. Azzopardi D. Alsaffar A. Tiquia-Arashiro S. M. (2018). Patterns of change in metabolic capabilities of sediment microbial communities along river and lake ecosystems. J. Inter. Microbiol. 2018:6234931. doi: 10.1155/2018/6234931, PMID: 29977299 PMC5994298

[ref109] Oh S. E. Hassan S. H. Joo J. H. (2009). Biosorption of heavy metals by lyophilized cells of *Pseudomonas stutzeri*. World J. Microbiol. Biotechnol. 25, 1771–1778. doi: 10.1007/s11274-009-0075-6

[ref110] Ojeda J. J. Dittrich M. (2012). “Fourier transform infrared spectroscopy for molecular analysis of microbial cells” in Microbial systems biology. Methods in molecular biology. ed. Navid A., vol. 881 (Totowa, NJ: Humana Press) 10.1007/978-1-61779-827-6_8 22639215

[ref111] Ojeda J. J. Romero-Gonzalez M. E. Bachmann R. T. Edyvean R. G. Banwart S. A. (2008). Characterization of the cell surface and cell wall chemistry of drinking water bacteria by combining XPS, FT-IR spectroscopy, modeling, and potentiometric titrations. Langmuir 24, 4032–4040. doi: 10.1021/la702284b 18302422

[ref112] Pagnucco G. Overfield D. Chamlee Y. Fortuna A. Sulaiman F. Farinas J.. (2023). Metal tolerance and biosorption capacities of bacterial strains isolated from an urban watershed. Front. Microbiol. 14:1278886. doi: 10.3389/fmicb.2023.1278886 37942073 PMC10630031

[ref113] Pardo R. Herguedas M. Barrado E. Vega M. (2003). Biosorption of cadmium, copper, lead and zinc by inactive biomass of *Pseudomonas putida*. Anal. Bioanal. Chem. 376, 26–32. doi: 10.1007/s00216-003-1843-z, PMID: 12734614

[ref114] Patel D. Gismondi R. Alsaffar A. Tiquia-Arashiro S. M. (2019). Applicability of API ZYM to capture seasonal and spatial variabilities in lake and river sediments. Environ. Technol. 40, 3227–3239. doi: 10.1080/09593330.2018.1468492 29683032

[ref115] Perkins D. L. Lovell C. R. Bronk B. V. Setlow B. Setlow P. Myrick M. L. Classification of endospores of Bacillus and Clostridium species by FT-IR reflectance microspectroscopy and autoclaving. IM S 2005- IEEE international workshop on measurement Systems for Homeland Security, contraband detection and personal safety. (2005). Orlando, FL. 81–87

[ref116] Pirutin S. K. Jia S. Yusipovich A. I. Shank M. A. Parshina E. Y. Rubin A. B. (2023). Vibrational spectroscopy as a tool for bioanalytical and biomonitoring studies. Int. J. Mol. Sci. 24:6947. doi: 10.3390/ijms24086947, PMID: 37108111 PMC10138916

[ref117] Plecha S. Hall D. Tiquia-Arashiro S. M. (2013). Screening for novel bacteria from the bioenergy feedstock switchgrass (*Panicum virgatum* L.). Environ. Technol. 34, 1895–1904. doi: 10.1080/09593330.2013.818701, PMID: 24350443

[ref118] Pomaranski E. Tiquia-Arashiro S. M. (2016). Butanol tolerance of carboxydotrophic bacteria isolated from manure composts. Environ. Technol. 37, 1970–1982. doi: 10.1080/09593330.2015.1137360, PMID: 26809187

[ref119] Portenier I. Waltimo T. Ørstavik D. Haapasalo M. (2005). The susceptibility of starved, stationary phase, and growing cells of *Enterococcus faecalis* to endodontic medicaments. J. Endod. 31, 380–386. doi: 10.1097/01.don.0000145421.84121.c8, PMID: 15851934

[ref120] Qin W. Amin S. A. Lundeen R. A. Heal K. R. Martens-Habbena W. Turkarslan S.. (2018). Stress response of a marine ammonia-oxidizing archaeon informs physiological status of environmental populations. ISME J. 12, 508–519. doi: 10.1038/ismej.2017.186, PMID: 29053148 PMC5776466

[ref121] Quilès F. Saadi S. Francius G. Bacharouche J. Humbert F. (2016). *In situ* and real time investigation of the evolution of a *Pseudomonas fluorescens* nascent biofilm in the presence of an antimicrobial peptide. Biochim Biophy Acta 1858, 75–84. doi: 10.1016/j.bbamem.2015.10.015, PMID: 26525662

[ref122] Rebuffo-Scheer C. A. Dietrich J. Wenning M. Scherer S. (2008). Identification of five Listeria species based on infrared spectra (FT-IR) using macrosamples is superior to a microsample approach. Anal. Bioanal. Chem. 390, 1629–1635. doi: 10.1007/s00216-008-1834-1, PMID: 18231779

[ref123] Ribeiro da Cunha B. Fonseca L. P. Calado C. R. C. (2020). Metabolic fingerprinting with Fourier-transform infrared (FT-IR) spectroscopy: towards a high-throughput screening assay for antibiotic discovery and mechanism-of-action elucidation. Meta 10:145. doi: 10.3390/metabo10040145, PMID: 32283661 PMC7240953

[ref124] Rolfe M. D. Rice C. J. Lucchini S. Pin C. Thompson A. Cameron A. D.. (2012). Lag phase is a distinct growth phase that prepares bacteria for exponential growth and involves transient metal accumulation. J. Bacteriol. 194, 686–701. doi: 10.1128/JB.06112-11, PMID: 22139505 PMC3264077

[ref125] Saranya K. Sundaramanickam A. Shekhar S. Meena M. Sathishkumar R. S. Balasubramanian T. (2018). Biosorption of multi-heavy metals by coral associated phosphate solubilising bacteria *Cronobacter muytjensii* KSCAS2. J. Environ. Manag. 222, 396–401. doi: 10.1016/j.jenvman.2018.05.083, PMID: 29870968

[ref126] Saulou C. Jamme F. Girbal L. Maranges C. Fourquaux I. Cocaign-Bousquet M.. (2013). Synchrotron FT-IR microspectroscopy of *Escherichia coli* at single-cell scale under silver-induced stress conditions. Anal. Bioanal. Chem. 405, 2685–2697. doi: 10.1007/s00216-013-6725-4, PMID: 23354575

[ref127] Scarsini M. Thurotte A. Veidl B. Amiard F. Niepceron F. Badawi M.. (2021). Metabolite quantification by Fourier transform infrared spectroscopy in diatoms: proof of concept on *Phaeodactylum tricornutum*. Front. Plant Sci. 12:756421. doi: 10.3389/fpls.2021.756421, PMID: 34858459 PMC8631545

[ref128] Schmitt J. Flemming H. C. (1998). FTIR-spectroscopy in microbial and material analysis. Int. Biodeter. Biodeg. 41, 1–11. doi: 10.1016/S0964-8305(98)80002-4

[ref129] Schmitt J. Nivens D. White D. C. Flemming H. C. (1995). Changes of biofilm properties in response to sorbed substances – an FTIR-ATR study. Wat. Sci. Technol. 32, 149–155. doi: 10.1016/0273-1223(96)00019-4

[ref131] Semeraro P. Giotta L. Talà A. Tufariello M. D'Elia M. Milano F.. (2023). A simple strategy based on ATR-FTIR difference spectroscopy to monitor substrate intake and metabolite release by growing bacteria. Spectrochimica Acta Part A 302:123031. doi: 10.1016/j.saa.2023.123031, PMID: 37392540

[ref132] Sharma M. Nandy A. Taylor N. Venkatesan S. V. Ozhukil Kollath V. Karan K.. (2020). Bioelectrochemical remediation of phenanthrene in a microbial fuel cell using an anaerobic consortium enriched from a hydrocarbon-contaminated site. J. Hazard. Mater. 389:121845. doi: 10.1016/j.jhazmat.2019.121845, PMID: 31862354

[ref133] Silverstein R. M. Webster F. X. Kiemle D. J. Spectrometric identification of organic compounds. Eight edition. (2014). New York: John Wiley & Sons. 464.

[ref134] Singhalage I. D. Seneviratne G. Madawala H. Manawasinghe I. S. (2018). Characterization of structural properties of fungal-bacterial biofilms by Fourier transform infrared spectroscopy. Ceylon J. Sci. 47, 77–83. doi: 10.4038/cjs.v47i1.7490

[ref135] Smirnova M. Tafintseva V. Kohler A. Miamin U. Shapaval V. (2022). Temperature-and nutrients-induced phenotypic changes of Antarctic green snow bacteria probed by high-throughput FTIR spectroscopy. Biology 11:890. doi: 10.3390/biology11060890, PMID: 35741411 PMC9220083

[ref136] Smith B. C. Infrared spectral interpretation: A systematic approach. (1998). CRC Press: Boca Raton, FL. 288.

[ref137] Smith B. C. Fundamentals of Fourier transform infrared spectroscopy. (2011). CRC Press, Boca Raton, FL. 207.

[ref138] Soares J. M. Guimarães F. E. G. Yakovlev V. V. Bagnato V. S. Blanco K. C. (2022). Physicochemical mechanisms of bacterial response in the photodynamic potentiation of antibiotic effects. Sci. Rep. 12:21146. doi: 10.1038/s41598-022-25546-y, PMID: 36476814 PMC9729225

[ref139] Spain O. Funk C. (2022). Detailed characterization of the cell wall structure and composition of nordic green microalgae. J. Agric. Food Chem. 70, 9711–9721. doi: 10.1021/acs.jafc.2c02783, PMID: 35894177 PMC9372998

[ref140] Stehfest K. Toepel J. Wilhelm C. (2005). The application of micro-FTIR spectroscopy to analyze nutrient stress-related changes in biomass composition of phytoplankton algae. Plant Physiol. Biochem. 43, 717–726. doi: 10.1016/j.plaphy.2005.07.001, PMID: 16122937

[ref141] Stuart B. Infrared spectroscopy: Fundamentals and applications. (2004). New York: John Wiley & Sons. p 224.

[ref142] Sundaram J. Park B. Hinton A. Jr. Yoon S. C. Windham W. R. Lawrence K. C. (2012). Classification and structural analysis of live and dead Salmonella cells using Fourier transform infrared spectroscopy and principal component analysis. J. Agric. Food Chem. 60, 991–1004. doi: 10.1021/jf204081g, PMID: 22257216

[ref143] Sundararaju S. Manjula A. Kumaravel V. Muneeswaran T. Vennila T. (2022). Biosorption of nickel ions using fungal biomass Penicillium sp. MRF1 for the treatment of nickel electroplating industrial effluent. Biomass Conv. Bioref. 12, 1059–1068. doi: 10.1007/s13399-020-00679-0

[ref144] Tang M. McEwen G. D. Wu Y. Miller C. D. Zhou A. (2013). Characterization and analysis of mycobacteria and gram-negative bacteria and co-culture mixtures by Raman microspectroscopy, FTIR, and atomic force microscopy. Anal. Bioanal. Chem. 405, 1577–1591. doi: 10.1007/s00216-012-6556-8, PMID: 23196750

[ref145] Tata A. Marzoli F. Cordovana M. Tiengo A. Zacometti C. Massaro A.. (2023). A multi-center validation study on the discrimination of *Legionella pneumophila* sg. 1, *Legionella pneumophila* sg. 2-15 and Legionella non-pneumophila isolates from water by FT-IR spectroscopy. Front. Microbiol. 14:1150942. doi: 10.3389/fmicb.2023.1150942 37125166 PMC10133462

[ref146] Tessaro L. Mutz Y. D. S. Andrade J. C. Aquino A. Belem N. K. R. Silva F. G. S.. (2023). ATR-FTIR spectroscopy and chemometrics as a quick and simple alternative for discrimination of SARS-CoV-2 infected food of animal origin. Spectrochim. Acta A Mol. Biomol. Spectrosc. 285:121883. doi: 10.1016/j.saa.2022.121883, PMID: 36126622 PMC9473138

[ref147] Tiquia S. M. (2008). Diversity of sulfate-reducing genes (dsrAB) in sediments from Puget Sound. Environ. Technol. 29, 1095–1108. doi: 10.1080/09593330802190608, PMID: 18942577

[ref148] Tiquia S. M. (2010a). Metabolic diversity of the heterotrophic microorganisms and potential link to pollution of the Rouge River. Environ. Pollut. 158, 1435–1443. doi: 10.1016/j.envpol.2009.12.035, PMID: 20106574

[ref149] Tiquia S. M. (2010b). Salt-adapted bacteria isolated from the Rouge River and potential for degradation of contaminants and biotechnological applications. Environ. Technol. 31, 967–978. doi: 10.1080/09593331003706226, PMID: 20662385

[ref150] Tiquia S. M. (2011). Extracellular hydrolytic enzyme activities of the heterotrophic microbial communities of the Rouge River: An approach to evaluate ecosystem response to urbanization. Microb. Ecol. 62, 679–689. doi: 10.1007/s00248-011-9871-2, PMID: 21611688

[ref151] Tiquia S. M. Chong S. C. Fields M. W. Zhou J. Oligonucleotide-based functional gene arrays for analysis of microbial communities in the environment. In: Kowalchuk G. G. Bruijn F. J. de Head I. M. Akkermans A. D. Elsas J. D. van, editors. Molecular microbial ecology manual. Kluwer Academic Publishers: Netherlands. (2004). p. 1743–1763.

[ref152] Tiquia S. M. Gurczynski S. Zholi A. Devol A. (2006a). Diversity of biogeochemical cycling genes from Puget Sound sediments using DNA microarrays. Environ. Technol. 27, 1377–1389. doi: 10.1080/09593332708618756, PMID: 17285943

[ref153] Tiquia S. M. Masson S. A. Devol A. (2006b). Vertical distribution of nitrite reductase genes (nirS) in continental margin sediments of the Gulf of Mexico. FEMS Microbiol. Ecol. 58, 464–475. doi: 10.1111/j.1574-6941.2006.00173.x, PMID: 17117989

[ref154] Tiquia S. M. Schleibak M. Schlaff J. Floyd J. C. Benipal B. Zakhem E.. (2008). Microbial community profiling and characterization of some heterotrophic bacterial isolates from river waters and shallow groundwater wells along the Rouge River, Southeast Michigan. Environ. Technol. 29, 651–663. doi: 10.1080/09593330801986998, PMID: 18702291

[ref155] Tiquia S. M. Wan H. C. Tam N. F. Y. (2002). Microbial population dynamics and enzyme activities during composting. Compost. Sci. Utilization 10, 150–161. doi: 10.1080/1065657X.2002.10702075

[ref156] Tiquia-Arashiro S. M. (2012) Molecular biological Technologies for Ocean Sensing. Springer Protocols Handbooks. Humana Press, Totowa, NJ. 307.

[ref157] Tiquia-Arashiro S. M. (2014). “Microbial CO metabolism” in Thermophilic Carboxydotrophs and their Applications in Biotechnology. Springer Briefs in Microbiology. SM Tiquia-Arashiro, editor. (Cham: Springer)

[ref158] Tiquia-Arashiro S. M. (2018). Lead absorption mechanisms in bacteria as strategies for lead bioremediation. Appl. Microbiol. Biotechnol. 102, 5437–5444. doi: 10.1007/s00253-018-8969-6, PMID: 29736824

[ref159] Tiquia-Arashiro S. M. (2019a). “Synthesis of metallic nanoparticles by halotolerant fungi” in Fungi in extreme environments: Ecological role and biotechnological significance. eds. Tiquia-Arashiro S. Grube M. (Cham: Springer)

[ref160] Tiquia-Arashiro S. M. (2019b). “Thermophilic fungi in composts: their role in composting and industrial processes” in Fungi in extreme environments: Ecological role and biotechnological significance. eds. Tiquia-Arashiro S. Grube M. (Cham: Springer)

[ref161] Tiquia-Arashiro S. M. Grube M. (2019). Fungi in extreme environments: Ecological role and biotechnological significance. Basel: Springer Nature Switzerland AG. 626.

[ref162] Tiquia-Arashiro S. M. Pant D. (2019). Microbial electrochemical technologies. CRC Press, Boca Raton, FL. 518.

[ref163] Tong C. Y. Derek C. J. C. (2021). Biofilm formation of benthic diatoms on commercial polyvinylidene fluoride membrane. Algal Res. 55:102260. doi: 10.1016/j.algal.2021.102260

[ref165] Tugarova A. V. Scheludko A. V. Dyatlova Y. A. Filip'echeva Y. A. Kamnev A. A. (2017). FTIR spectroscopic study of biofilms formed by the rhizobacterium *Azospirillum brasilense* Sp245 and its mutant *Azospirillum brasilense* Sp245. 1610. J. Mol Struc. 1140, 142–147. doi: 10.1016/j.molstruc.2016.12.063

[ref166] Wang L. Fan D. Chen W. Terentjev E. M. (2015). Bacterial growth, detachment, and cell size control on polyethylene terephthalate surfaces. Sci. Rep. 5:15159. doi: 10.1038/srep15159, PMID: 26464114 PMC4604555

[ref167] Wang Y. Jiang Z. Lai Z. Yuan H. Zhang X. Jia Y.. (2021). The self-adaption capability of microalgal biofilm under different light intensities: photosynthetic parameters and biofilm microstructures. Algal Res. 58:102383. doi: 10.1016/j.algal.2021.102383

[ref168] Wang Y. D. Li X. L. Hu J. Lü J. H. (2019). Synchrotron infrared spectral regions as signatures for foodborne bacterial typing. Nucl. Sci. Techniques 30:25. doi: 10.1007/s41365-019-0554-x

[ref169] Wang H. Yang Q. Li D. Wu J. Yang S. Deng Y.. (2023). Stable isotopic and metagenomic analyses reveal microbial-mediated effects of microplastics on sulfur cycling in coastal sediments. Environ. Sci. Technol. 57, 1167–1176. doi: 10.1021/acs.est.2c06546, PMID: 36599128

[ref170] Watanabe R. Sugahara A. Shinzawa H. Yamane S. Nakamura S. Sato H.. (2023). Photodegradation behavior of polyethylene terephthalate analyzed by MALDI-TOFMS and ATR-FTIR microscopic analysis in combination with two-trace two-dimensional (2T2D) correlation mapping. Polym. Degr. Stabil. 208:110246. doi: 10.1016/j.polymdegradstab.2022.110246

[ref171] Weber A. Hoplight B. Ogilvie R. Muro C. Khandasammy S. R. Pérez-Almodóvar L.. (2023). Innovative vibrational spectroscopy research for forensic application. Anal. Chem. 95, 167–205. doi: 10.1021/acs.analchem.2c05094, PMID: 36625116

[ref172] Wenning M. Scherer S. (2013). Identification of microorganisms by FTIR spectroscopy: perspectives and limitations of the method. Appl. Microbiol. Biotechnol. 97, 7111–7120. doi: 10.1007/s00253-013-5087-3, PMID: 23860713

[ref173] Wilson R. M. Zayed A. A. Crossen K. B. Woodcroft B. Tfaily M. M. Emerson J.. (2021). Functional capacities of microbial communities to carry out large scale geochemical processes are maintained during ex situ anaerobic incubation. PLoS One 16:e0245857. doi: 10.1371/journal.pone.0245857, PMID: 33630888 PMC7906461

[ref174] Wu W. Gu B. Fields M. W. Gentile M. Ku Y.-K. Yan H.. (2005). Uranium (VI) reduction by denitrifying biomass. Biorem. J. 9, 49–61. doi: 10.1080/10889860590929628

[ref175] Yang H. Shi H. Feng B. Wang L. Chen L. Alvarez-Ordóñez A.. (2023). Protocol for bacterial typing using Fourier transform infrared spectroscopy. STAR Protoc. 4:102223. doi: 10.1016/j.xpro.2023.102223, PMID: 37061919 PMC10130498

[ref176] Yu P. (2005). Applications of hierarchical cluster analysis (CLA) and principal component analysis (PCA) in feed structure and feed molecular chemistry research, using synchrotron-based Fourier transform infrared (FTIR) microspectroscopy. J. Agric. Food Chem. 53, 7115–7127. doi: 10.1021/jf050959b, PMID: 16131119

[ref177] Yu C. Irudayaraj J. (2005). Spectroscopic characterization of microorganisms by Fourier transform infrared microspectroscopy. Biopolymers 77, 368–377. doi: 10.1002/bip.20247, PMID: 15700299

[ref178] Zada S. Xie J. Yang M. Yang X. Sajjad W. Rafiq M.. (2021). Composition and functional profiles of microbial communities in two geochemically and mineralogically different caves. Appl. Microbiol. Biotechnol. 105, 8921–8936. doi: 10.1007/s00253-021-11658-4, PMID: 34738169

[ref179] Zarnowiec P. Lechowicz Ł. Czerwonka G. Kaca W. (2015). Fourier transform infrared spectroscopy (FTIR) as a tool for the identification and differentiation of pathogenic bacteria. Curr. Med. Chem. 22, 1710–1718. doi: 10.2174/0929867322666150311152800, PMID: 25760086

[ref180] Zeroual W. Choisy C. Doglia S. M. Bobichon H. Angiboust J. F. Manfait M. (1994). Monitoring of bacterial growth and structural analysis as probed by FT-IR spectroscopy. Biochimica et Biophysica Acta-Mol Cell Res 1222, 171–178. doi: 10.1016/0167-4889(94)90166-X, PMID: 8031853

[ref181] Zhang J. Su P. Chen H. Qiao M. Yang B. Xu Z. (2023). Impact of reactive oxygen species on cell activity and structural integrity of gram-positive and gram-negative bacteria in electrochemical disinfection system. Chem. Eng. J. 451:138879. doi: 10.1016/j.cej.2022.138879

[ref9002] Zhao H. Parry R. L. Ellis D. I. Griffith G. W. Goodacre R. (2006). The rapid differentiation of Streptomyces isolates using Fourier transform infrared spectroscopy. Vibrational Spectroscopy. 40:213–218. doi: 10.1016/j.vibspec.2005.09.006

[ref182] Zlotnikov I. D. Ezhov A. A. Vigovskiy M. A. Grigorieva O. A. Dyachkova U. D. Belogurova N. G.. (2023). Application prospects of FTIR spectroscopy and CLSM to monitor the drugs interaction with bacteria cells localized in macrophages for diagnosis and treatment control of respiratory diseases. Diagnostics 13:698. doi: 10.3390/diagnostics13040698, PMID: 36832185 PMC9954918

