# Corrigendum: Applications of Fourier Transform-Infrared spectroscopy in microbial cell biology and environmental microbiology: advances, challenges, and future perspectives

**DOI:** 10.3389/fmicb.2023.1342406

**Published:** 2023-12-13

**Authors:** Amin Kassem, Lana Abbas, Oliver Coutinho, Somie Opara, Hawraa Najaf, Diana Kasperek, Keshav Pokhrel, Xiaohua Li, Sonia Tiquia-Arashiro

**Affiliations:** ^1^Department of Natural Sciences, University of Michigan-Dearborn, Dearborn, MI, United States; ^2^Department of Mathematics and Statistics, University of Michigan-Dearborn, Dearborn, MI, United States

**Keywords:** Fourier transform infrared spectroscopy, cellular structures, metabolic activities, bacteria, microbial stress, cell membrane, population dynamics

In the published article, there was an error in the author list, and the sequence of authors was incorrectly listed. The corrected author list appears below.

“Amin Kassem^1^, Lana Abbas^1^, Oliver Coutinho^1^, Somie Opara^1^, Hawraa Najaf^1^, Diana Kasperek^1^, Keshav Pokhrel^2^, Xiaohua Li^1^ and Sonia Tiquia-Arashiro^1^^*^”

The authors apologize for this error and state that this does not change the scientific conclusions of the article in any way. The original article has been updated.

